# Expanded applications of bioluminescence microscopy with phasor analysis

**DOI:** 10.1016/j.crmeth.2026.101344

**Published:** 2026-03-30

**Authors:** Lila P. Halbers, Caroline K. Brennan, Lorenzo Scipioni, Giulia Tedeschi, Zachary R. Torrey, Kshitij Parag-Sharma, Bryan Labra, Christoph Gohlke, Antonio L. Amelio, Michelle A. Digman, Jennifer A. Prescher

**Affiliations:** 1 Department of Pharmaceutical Sciences, University of California, Irvine, Irvine, CA, USA; 2 Laboratory for Fluorescence Dynamics, Department of Biomedical Engineering, University of California, Irvine, Irvine, CA, USA; 3 Department of Chemistry, University of California, Irvine, Irvine, CA, USA; 4 Graduate Curriculum in Cell Biology & Physiology, Biological & Biomedical Sciences Program, UNC School of Medicine, The University of North Carolina at Chapel Hill, Chapel Hill, NC, USA; 5 Lineberger Comprehensive Cancer Center, The University of North Carolina at Chapel Hill, Chapel Hill, NC, USA; 6 Department of Otolaryngology/Head and Neck Surgery, University of North Carolina at Chapel Hill, Chapel Hill, NC, USA; 7 Department of Tumor Microenvironment and Metastasis, H. Lee Moffitt Cancer Center & Research Institute, Tampa, FL, USA; 8 Department of Head and Neck-Endocrine Oncology, H. Lee Moffitt Cancer Center & Research Institute, Tampa, FL, USA; 9 Department of Molecular Biology and Biochemistry, University of California, Irvine, Irvine, CA, USA

**Keywords:** bioluminescence, imaging, spectral phasor, luciferase, luciferin, biosensor

## Abstract

Bioluminescence is a powerful technique for monitoring biological processes *in vitro* and *in vivo*. A variety of bioluminescent reporters have been developed that span the visible spectrum, but simultaneous detection remains challenging due to substantial spectral overlap. To address this issue, we previously developed bioluminescent phasor analysis to resolve spectrally similar probes. In the current work, we characterize the phasor outputs of several common luciferase reporters and expand the method for distinguishing complex mixtures in tissues. We further apply bioluminescent phasor for imaging biosensors in multidimensional models. Subtle changes in bioluminescent outputs were readily resolved, enabling previously indistinguishable changes in analyte-dependent spectra to be detected. Collectively, this work broadens the utility of bioluminescent phasor imaging for multiplexed imaging applications.

## Highlights

• We present expanded applications of bioluminescent phasor imaging
• Multiple luciferase reporters are readily distinguishable in multidimensional models
• Bioluminescent biosensors can be resolved at the single-cell level

## Motivation

Continuous, multiplexed imaging remains a major challenge for biological studies. Collections of well-resolved fluorescent reporters are available for multicomponent visualization, but their requirement for excitation light can limit the overall duration and scope of the experiment. Bioluminescence can offer a more biocompatible alternative, but a lack of optical methods to resolve multiple probes has historically stymied progress. We previously developed a platform—bioluminescent phasor—to fill the void in imaging capabilities. In the current work, we evaluate a more comprehensive set of reporters for multiplexed visualization and implement bioluminescent phasor in cells, tissues, and other complex environments, setting the stage for multiplexed imaging across a broader range of targets.

## Graphical abstract

**Figure:**
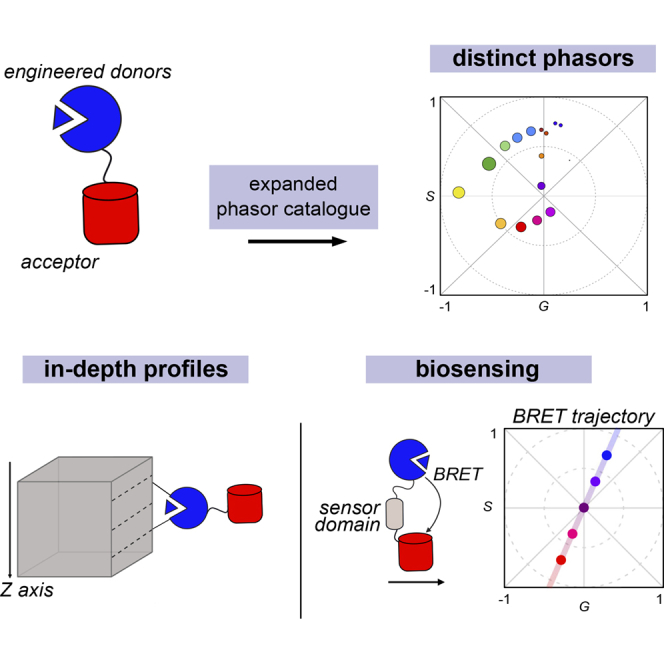

## Teaser

Halbers et al. advance multiplexed bioluminescence imaging by employing phasor analysis with an expanded set of luciferase reporters. Through applications to imaging biosensors, they demonstrate subtle resolutions in light output that can broaden the scope of detectable targets.

## Introduction

Bioluminescence is a useful technology for imaging in tissues and whole organisms.^1^^,^^2^^,^^3^^,^^4^ The method relies on light production from the luciferase-mediated oxidation of small-molecule luciferins. No excitation light is required, making bioluminescence exceptionally well suited for imaging in opaque tissues and over long time periods. Indeed, the technique has long been used to track changes in gene expression,^5^ disease onset,^6^ and other biological processes *in vivo*.^7^^,^^8^^,^^9^ The number and types of applications continue to grow, thanks to recent advances in luciferase and luciferin substrate designs that are enabling ever more sensitive readouts at the whole-organism (macro) scale.^10^^,^^11^^,^^12^^,^^13^^,^^14^^,^^15^

Many of the advantages provided by bioluminescent reporters can now, in principle, extend to the microscale. Improved detectors and enzymes have overcome some of the historic challenges associated with luciferase imaging, namely dim emission. Faster-acting enzymes and more sensitive cameras have dramatically shortened the necessary acquisition times, resulting in higher resolution images. In fact, many applications using the engineered enzyme NanoLuc match the spatiotemporal resolution achieved with traditional fluorescent reporters.^16^^,^^17^ NanoLuc is two to three orders of magnitude brighter than traditional luciferases, enabling routine imaging of single-cell and organelle structures.^18^^,^^19^^,^^20^^,^^21^^,^^22^

Multicolored reporters have also been generated via NanoLuc fusion to fluorescent proteins. These constructs emit different wavelengths of light via bioluminescent Förster resonance energy transfer (BRET),^23^ with the luciferase serving as the donor and the fluorescent protein serving as the acceptor. Notable BRET reporters include the enhanced Nano-lantern (NL) series^19^ and LumiFluors,^24^^,^^25^^,^^26^ among others.^14^^,^^27^^,^^28^ BRET reporters have been further engineered to sense metabolites and other biochemical features.^20^^,^^29^^,^^30^ In these cases, the reporters typically exhibit a change in optical output (e.g., BRET ratio) in response to the target of interest. The resulting shifts in emission can then provide quantitative readouts on the underlying biomolecule or process.

Despite the growing toolkit of BRET-based reporters and readouts, challenges remain with multiplexed detection. This is due, in part, to the broad, overlapping emission profiles of bioluminescent reporters. Typical emission spectra for these reporters are difficult, if not impossible, to resolve with filter-based methods alone. The problem is further exacerbated with bioluminescent sensors, which often require discerning subtle differences in the relative intensities of overlapping peaks. Residual emission from the donor can further drown out signals from the acceptor, limiting the sensitivity of detection and overall dynamic range of signals. Several efforts to enable more facile multicomponent bioluminescence have been pursued, including the development of orthogonal enzyme-substrate pairs.^31^ While effective in some contexts, this approach involves sequential imaging steps and may present challenges for continuous, real-time imaging applications. Multiple bioluminescent probes can be registered on RGB and related cameras,^32^ but these can limit the amount of light captured and require spatial separation of each emitter.

To address the need for more facile multiplexing of luminescent reporters, we developed an approach, termed bioluminescent phasor.^33^ This method uses an optical Fourier transform to convert emission spectra to phasor coordinates (*G,S*) in a pixelwise manner. The emission is thus represented as a point on a phasor plot. The phasor signature encompasses the entire spectrum for a given reporter, so even small changes (10 nm) in emission register as unique signals on the plot. Differences in BRET efficiency among similar donor-acceptor pairs can also be readily discerned with high precision. Additionally, phasor transformation takes place in real time, without the need for filter switching. This is an important feature for many biosensing applications, especially when dealing with low-light emitters that can decay over time. The phasor method relies on three channels, one of which is unfiltered, and the other two provide higher transmission than conventional RGB cameras, enabling more efficient photon detection. The method can also be used with any single-color camera. We previously achieved efficient multiplexing with seven reporters *in vitro* and *in cellulo*, and we were able to track multiple subcellular features over time using different cameras.^33^

**Figure 1:**
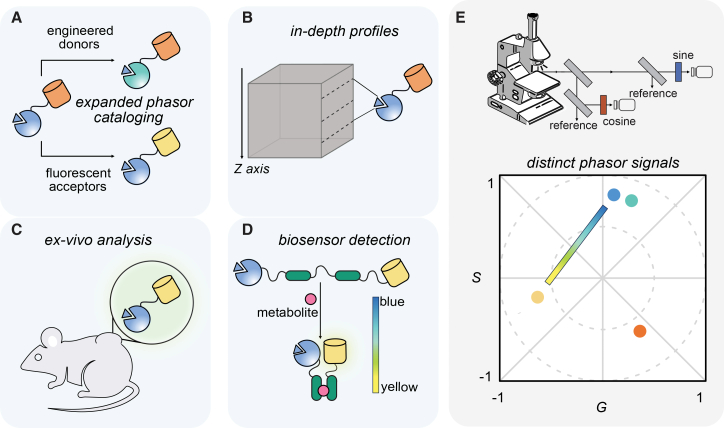
Expanding the scope of bioluminescent phasor imaging (A) Schematic of BRET reporters comprising alternative structural features. (B) Schematic of profiling bioluminescent phasor readouts at various depths. (C) Schematic of analyzing tissue samples via bioluminescent phasor analysis. (D) Schematic of metabolite detection with bioluminescent probes. (E) Schematic of bioluminescent phasor imaging rig and readouts of multiple reporters. Unique phasors occupy distinct locations on the phasor plot (colored circles). Profiles can be established using multiple methods. If a pixel contains two emission components, the phasor position lies between the two independent components (represented by the gradient between yellow and blue circles).

Here, we build upon bioluminescent phasor technology and provide a more detailed assessment of its applications for dynamic, multiplexed imaging (Figure 1). We first catalog the spectral phasor outputs of an expanded set of bioluminescent reporters. New combinations of luciferases and luciferins are identified that cannot be discriminated by wavelength alone (Figure 1A). We further examine multiplexed detection in tissues and other complex environments, setting the stage for true micro-to-macro imaging studies (Figures 1B and 1C). Last, we evaluate bioluminescent phasor in conjunction with two common BRET sensors (Figure 1D). Readouts were possible at the single-cell level, both over time and in tissue-like environments. Overall, the collection of probes and applications we establish here broaden the scope of bioluminescent phasor technology for imaging molecular and cellular features (Figure 1E).

## Results

### Bioluminescent phasor characterization of BRET reporters

**Figure 2:**
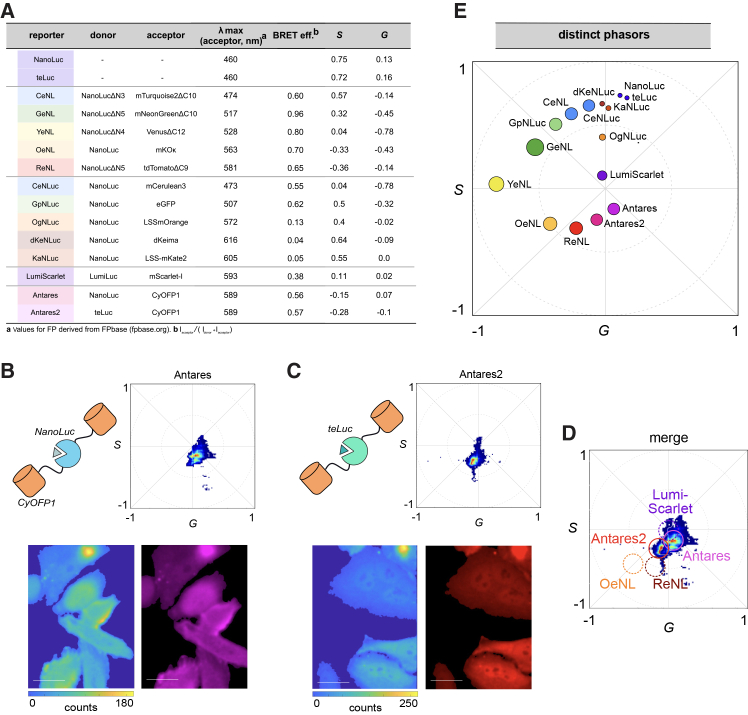
Bioluminescent phasor signatures capture distinct structural and spectral features (A) Table of reporters used in this study. Distinct luciferase fusions exhibit different BRET efficiencies and unique phasor outputs (represented by *S*, *G* coordinates) in the presence of furimazine (Fz). (B and C) Bioluminescent phasor signatures of Antares (B) and Antares2 (C). HeLa cells were transiently transfected with plasmids encoding the reporters. The cells were then treated with Fz (20 μM) ∼36 h post transfection. Images were acquired using the bioluminescent phasor microscope with a 20× air objective and 10 s/frame integration time. A total of 20 frames were collected for each sample and the phasor locations were computed. Each pixel in the resulting image was color-coded according to the phasor signature. Scale bars, 25 μm. (D) The phasor signatures of Antares and Antares2 are distinct from those of other red-shifted BRET reporters. (E) Compiled phasor signatures of common BRET reporters. The size of each circle represents the calculated BRET ratio.

Ideal probe combinations for bioluminescent phasor are not intuitive from emission data alone, as the spectral shape plays an important role in the readouts. We previously identified phasor signatures for the enhanced NLs (eNLs),^19^^,^^33^ a bright and well-established set of luciferases for imaging studies. In the current work, we aimed to expand our analysis of microscopy-ready luciferases. These tools span a range of spectral outputs and can be advantageous for different experiments, depending on the dynamic range, cell type, or substrate features required. We first cataloged the spectral phasors (*G,S*) of additional enzymes that use furimazine (Fz) in the light emitting reaction (Figure 2A). Included in this group were the LumiFluors, featuring NanoLuc fused to mCerulean3 (CeNLuc), eGFP (GpNLuc), or LSSmOrange (OgNLuc).^24^ These probes have been applied to imaging tumorigenesis *in vivo*^24^ and various optogenetics pursuits.^34^ HeLa cells were engineered to express each LumiFluor, treated with Fz, and then analyzed via bioluminescent phasor (Figure S1). Each LumiFluor could be readily distinguished from previously reported eNLs, despite their similar emission profiles (Δλ_max_ ∼10 nm, Figures S1A–S1M).^33^ OgNLuc could also be readily resolved from LumiScarlet. These luciferases are attractive for *in vivo* imaging owing to their generation of more tissue-penetrant light. Other LumiFluors with red-emitting fluorescent acceptors, dKeNLuc (dKeima-NLuc) and KaNLuc (LSSmKate-NLuc),^26^ also generated unique phasor signatures (Figures S2A–S2E), albeit with lower BRET efficiency, and are amenable to future multiplexed imaging studies.

We further characterized variants of Antares, a NanoLuc fusion comprising two CyOFP proteins.^27^ Antares exhibits among the most red-shifted and superior detectable emission of known BRET reporters.^35^ As a consequence, this probe has been widely used for *in vivo* applications demanding extreme sensitivity, including CAR-T cell tracking and non-invasive calcium imaging in the brain.^12^ A related reporter, Antares2, was developed for deep tissue imaging.^13^ This fusion comprises a NanoLuc mutant (teLuc) that catalyzes light emission with diphenylterazine (DTZ), a red-shifted luciferin analog of Fz (Figures S2F and S2G). Although Antares and Antares2 comprise the same fluorescent acceptor (CyOFP), they exhibit unique BRET signatures and thus register distinct signals on the bioluminescent phasor plot (Figures 2B and 2C). Excitingly, these signatures are also readily distinguishable from other red-shifted probes (Figure 2D).^33^

With the expanded set of phasor measurements, we generated a comprehensive dataset to guide bioluminescent probe selection. Each luciferase reporter is shown in Figure 2E, with the position of the phasor reflecting the color/spectral output and the size indicating BRET efficiency. The reporters occupying the more peripheral positions (e.g., YeNL and GeNL) exhibit higher overall efficiencies, and, thus, the spectral output is dominated by the color of the acceptor. The BRET efficiencies for reporters occupying the center of the plot (e.g., LumiScarlet) contain a larger fraction of the donor (i.e., NanoLuc). Reporters that are positioned further apart in the phasor space are most readily separated and attractive for multiplexed readouts.

The efficiencies determined via phasor analysis are mostly in agreement with those achieved with the conventional spectral methods (Tables S1 and S2). These latter approaches involve transforming measured emission spectra into spectral phasor coordinates, as implemented in the open-source Python library PhasorPy (version 0.6, https://zenodo.org/records/15717305), enabling a direct comparison with phasor-based analysis. Some differences were observed, though, particularly with the most red-emitting bioluminescent probes. These inconsistencies are likely due to differences in acquisition schemes, which are most pronounced for >650 nm light.

### Bioluminescent phasor imaging through depth

**Figure 3:**
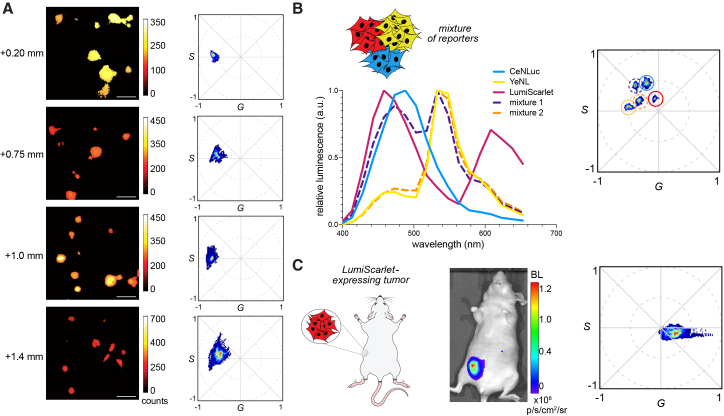
Bioluminescent phasor imaging through depth (A) Bioluminescent phasor images (left) and analysis plots (right) for YeNL-expressing HeLa cells embedded in a collagen matrix (2.0 mg/mL). Media containing furimazine (Fz, 50 μM) was added to the top of the sample, and light emission was recorded with the bioluminescent phasor microscope at various distances from the bottom of the slide. “Counts” represent the intensity above background. A total of 20 frames were collected per slice, and the phasor location was computed. Scale bars, 100 μm. (B) Excised subcutaneous tumors comprising UM-HMC-1 cells were analyzed. The cells expressed either CeNLuc, YeNL, or LumiScarlet, and mixtures of these reporter cells were also included. Spectra were measured on a luminometer (left), and phasor signatures were acquired with bioluminescent phasor (right). (C) LumiScarlet-expressing MDA-MB-231 cells implanted in a mouse were imaged using an EM-CCD camera (left), followed by the bioluminescent phasor microscope (right).

Bioluminescent phasor can be used to image cells in more complex environments. We previously showcased its utility for imaging tumor spheroids,^33^ and in the current study, we evaluated its performance in more tissue-like environments. HeLa cells stably expressing YeNL were embedded in collagen matrix at various depths (Figure 3A). Luciferase substrate was supplied, allowed to diffuse to mimic biodistribution, and images were acquired and registered with bioluminescent phasor. As shown in Figure 3A, cells could be visualized throughout the majority of the collagen stack, up to a maximum depth of ∼1.4 mm. Similar results were obtained using YeNL-expressing MDA-MB231 cells (Figure S3A). In both cases, phasor outputs broadened with the increasing depth. This outcome is expected due to additional light scattering through heterogeneous material. Notably, though, the center maximum of the phasor signature did not change in either case.

We further showcased bioluminescent phasor for imaging resected subcutaneous xenograft tumors. UM-HMC-1 mucoepidermoid carcinoma cells expressing distinct reporters—CeNLuc, YeNL, or LumiScarlet—were implanted in mice, both individually and in various combinations (mixtures 1 and 2). The three luciferases are well resolved on the phasor plot and provided a good test platform for the approach. The excised tumors were injected with Fz and imaged using the microscope (Figure 3B), and distinct phasor outputs were observed. The phasor positions matched the composition of the tissue samples (as determined by fluorescence measurements for the reporters, Figure S3B). For example, as shown in Figure 3B, the phasor readouts for the tissues comprising mixtures of reporters were located between the phasor positions for the individual reporters.

Bioluminescent phasor analysis is also compatible with *in vivo* imaging studies. As an initial demonstration, we examined LumiScarlet-expressing tumors in mouse models. LumiScarlet emits a large percentage of tissue-penetrant (red) light, making it well suited for use *in vivo*.^14^ LumiScarlet-expressing MDA-MB-231 cells were implanted in the flank of an athymic nude mouse. Once tumors were palpable, the mice were administered Fz and imaged via traditional bioluminescence imaging (using an EM-CCD camera) to confirm reporter expression. The same mice were then analyzed on the bioluminescent phasor microscope, and photon outputs registered on the phasor plot (Figure 3C). The overall phasor position shifted from LumiScarlet cells in isolation, perhaps due to differences in environmental factors. For example, the spectral outputs of both fluorescent proteins and luciferases can be impacted by oxidative stress.^36^ We observed a similar effect upon treating a red-emitting BRET reporter (ReNL) with 0.5%–1% H_2_O_2_ (Figure S3C).

We also noticed that the phasor fingerprint was broader than the readout from isolated LumiScarlet cells, similar to other samples imaged in more complex environments. To better understand the effects of depth and light scattering on bioluminescent phasor, an *in vitro* model was developed. Cells expressing unique bioluminescent reporters were plated on top of varying levels of collagen (0–1 mm), treated with Fz, then imaged with bioluminescent phasor. For blue (NanoLuc)-emitting cells, the emission spectra broadened with the increasing collagen thickness (Figure S4A), consistent with increased light scattering. Phasor analysis corroborated this result, with broadened phasor outputs observed with the increasing depth (Figure S4B). Yellow (YeNL)- and red (LumiScarlet)-emitting cells were subjected to similar analyses (Figure S4C). These cells exhibited reduced broadening with the increased collagen depth, as these colors of light are less scattered.

### Tracking proteolysis in 3D models with bioluminescent phasor analysis

Bioluminescent phasor can provide direct readouts on BRET efficiency, the key parameter for many microscale biosensing applications. A handful of bioluminescent sensors have been developed for monitoring G protein-coupled receptor (GPCR) activation and other activities.^37^^,^^38^^,^^39^ The classic design features a luciferase donor and fluorescent protein acceptor flanking a target binding domain or other sensing motif. The biomolecule or activity of interest changes the proximity of the donor and acceptor, resulting in a detectable change in BRET. The closer the pair, the more the BRET is observed; the farther apart, only partial or no BRET is observed. The spectral changes can be quite subtle and difficult to register, though, limiting the widespread adoption of bioluminescence for biosensing at the microscale.

Bioluminescent phasor imaging can report on small spectral differences common to BRET reporters, and because the technology takes the full spectrum of emission into account, residual signal from a donor or variable resonance energy transfer is not detrimental. In fact, the position of the phasor for a given sensor—thresholded by the phasor positions for the luciferase donor and fluorescent acceptor—provides a direct readout on BRET efficiency.^33^ Such refined measurements instantly report on the levels of a target biomolecule or activity and can provide critical insights into biomolecule dynamics at a single-cell level. Heterogeneity across a cell population can also be readily visualized, and the readouts can be acquired continuously, without concern for phototoxicity or photobleaching.

**Figure 4:**
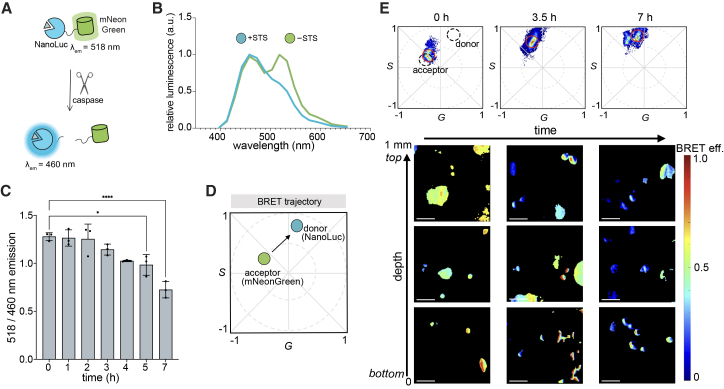
Caspase biosensing through depth (A) Caspase-9-responsive BRET (C9-BRET) sensor for reporting on cellular caspase-9 activity. Cleavage of the sensor results in a green-to-blue shift in emission. (B) Emission spectra of HeLa cells expressing C9-BRET in the presence and absence of staurosporine (STS, 5 μM). (C) Cleavage of C9-BRET monitored over time in the presence of STS (5 μM). The ratio of green (518 nm) to blue (460 nm) is shown, with *p* < 0.15 at 5 h and *p* < 0.001 at 7 h. *p* values (95% confidence interval) were calculated using Tukey’s multiple comparisons test. Data are presented as the mean ± SD for *n* = 3 replicates. (D) Schematic of C9-BRET biosensing via bioluminescent phasor analysis. (E) HeLa cells expressing C9-BRET embedded in a collagen matrix (2.0 mg/mL). Media containing furimazine (Fz, 50 μM) and STS (50 μM) was added to the top of the sample, and light emission was recorded over time and at various distances and imaged from the bottom (0 mm) and middle (0.5 mm), to top (1 mm) of the slide. A total of 20 frames were collected. BRET efficiency (eff.) represents the change in color (ratio of green: blue) over time. Values were scaled from 0 to 1.0. Representative phasor locations from the 0.5 mm (middle) plane at different time points were computed and are shown above the images. Scale bars, 100 μm.

To examine bioluminescent phasor analysis in the context of biosensing, we pursued two models. First, we employed a caspase-9-responsive BRET (C9-BRET) sensor (Figure 4A).^40^ This tool comprises NanoLuc (donor) linked to mNeonGreen (acceptor) via a caspase-9 recognition site. In the absence of the target protease, NanoLuc and mNeonGreen remain in proximity, and BRET (primarily green emission) is observed upon luciferin addition. However, upon caspase activation, the sensor is cleaved and NanoLuc emission (blue) is observed upon luciferin addition. We initially evaluated the sensor by using bulk cell luminometer measurements. C9-BRET was expressed in HeLa cells, and cultures were treated with staurosporine (STS), a kinase inhibitor that induces caspase activity. An increase in NanoLuc emission (460 nm) was observed, along with a decrease in mNeonGreen emission (518 nm), consistent with the expected cleavage of the sensor and overall decrease in BRET efficiency (Figure 4B).^40^ Cultures expressing the sensor were further subjected to STS treatment and monitored over time. The largest change in BRET efficiency was observed 5–7 h after incubation (Figure 4C). We hypothesized that C9-BRET (and thus caspase activity) could be readily monitored by bioluminescent phasor, with the phasor position moving along a trajectory from the acceptor to donor (Figure 4D). We further evaluated the readout in a 3D matrix to showcase the generality and modularity of bioluminescent phasor for protease sensing. Cells were embedded at varying depths in a collagen matrix and treated with or without STS, followed by Fz at each time point. Images were acquired over time and in different planes. A shift in the phasor signature from mNeonGreen to NanoLuc was observed in all instances, correlating with caspase-9 activity (Figure 4E). No shift was detected in the absence of STS (Figure S5). These results suggest that BRET biosensing is compatible with serial, long-term phasor imaging in tissue-like environments.

### Imaging changes in calcium events with bioluminescent phasor

**Figure 5:**
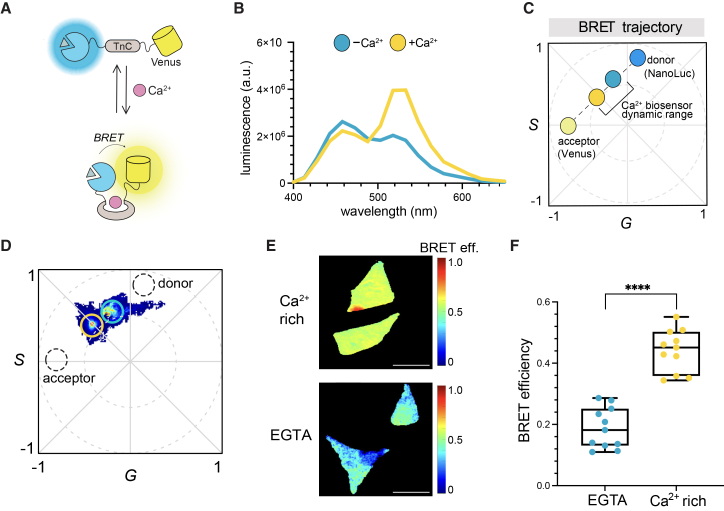
BRET efficiency phasor detection using calcium biosensing (A) CaFluxVTN sensor for reporting on cellular Ca^2+^ levels. Ca^2+^ binding to troponin C (TnC) induces a conformational change, bringing NLuc in proximity to Venus. In the presence of the furimazine (Fz) and Ca^2+^, more yellow light is produced. (B) Spectra of HeLa cells expressing CaFluxVTN sensor in the presence (+) of Ca^2+^ (500 μM) or no additional (−) Ca^2+^ (endogenous levels). (C) Schematic of BRET trajectory and dynamic range of CaFluxVTN sensor on bioluminescent phasor. (D) Phasor signatures of HeLa cells expressing CaFluxVTN in the presence of EGTA (10 μM) (yellow highlight) or Ca^2+^ (500 μM) (blue highlight). The positions of the donor and acceptor (in isolation) are shown for reference. A total of 20 frames were collected per slice, and the phasor locations were computed. (E) Single-cell BRET analyses using *G* and *S* coordinates derived from the corresponding phasor plots. BRET efficiency values were scaled from 0 to 1.0. Scale bars, 25 μm. (F) BRET efficiency measurements from single cells in (E). *p* < 0.0001. *P* (95% confidence interval) values were calculated using a two-sided *t* test. Data are presented as the mean ± SD for *n* = 10 cells.

The second biosensor examined was CaFluxVTN, a reporter of intracellular calcium levels (Figure 5A). CaFluxVTN comprises a Ca^2+^-sensitive troponin-C (TnC) sequence sandwiched between NanoLuc and Venus fluorescent protein.^41^ In the absence of Ca^2+^, NanoLuc emission is primarily observed. In the presence of excess Ca^2+^, binding to TnC is observed, resulting in a conformational change that brings NanoLuc in proximity to Venus. The end result is an increase in BRET (yellow emission) upon luciferase substrate administration.

HeLa cells were engineered to express CaFluxVTN and evaluated upon treatment with excess Ca^2+^ or EGTA, a known Ca^2+^ chelator (Figures 5B and S6). Emission spectra were acquired and confirmed that the sensor was active, with increased yellow emission at higher Ca^2+^ levels, and increased blue emission at lower levels. We then proceeded to examine the biosensing platform in more complex imaging experiments. We hypothesized that changes in Ca^2+^ levels (and thus changes in BRET outputs) could be readily tracked via bioluminescent phasor analysis (Figure 5C). Cells were exposed to exogenous Ca^2+^ or EGTA and then imaged (Figure 5D). Distinct phasor locations were observed in response to high or low Ca^2+^ stimulation. The BRET efficiencies of single cells were then analyzed using their phasor spectral coordinates (Figures 5E and 5F). Higher Ca^2+^ concentrations were required to elicit a spectral response from CaFluxVTN compared to those mentioned in previous reports (Figure S6A),^41^^,^^42^ likely due to differences in sensor expression or assay conditions. Nevertheless, Ca^2+^-dependent changes in signals could be robustly tracked over a wide range (0–15 mM), as shown by both spectral and phasor analyses (Figures S6B–S6E). BRET ratios calculated from the emission spectra exhibited sigmoidal behavior across this concentration range (Figure S6B). In addition, spectral decomposition (Figures S6C–S6D) revealed the dynamic contributions of NanoLuc and Venus. These data were subsequently transformed into phasor space (Figure S6E) to establish a continuous mapping between Ca^2+^ concentration, BRET efficiency, and phasor position. Together, these results define a spectral and phasor coordinate map that links Ca^2+^ concentration to BRET signal characteristics, providing a reference framework for interpreting the dynamic sensor responses observed in live cells (Figures 5D–5F).

**Figure 6:**
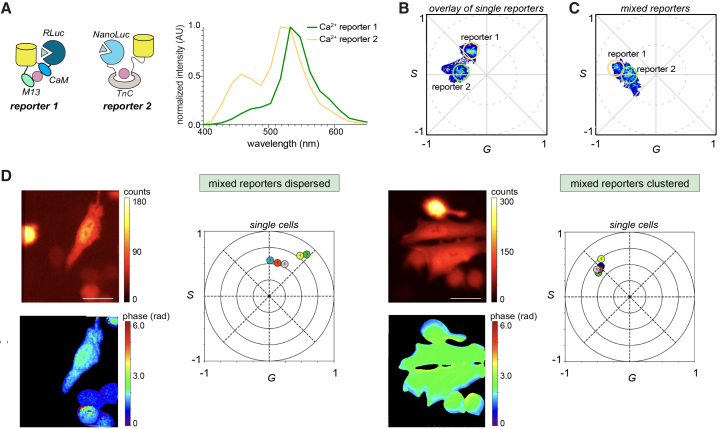
Multiplexed calcium biosensing in a mixed cell population (A) Spectra of HEK cells expressing Nano-Lantern(Ca^2+^) sensor (Ca^2+^ reporter 1 in green) and HeLa cells expressing CaFluxVTN (Ca^2+^ reporter 2 in yellow) in the presence of furimazine (Fz) or coelenterazine (CTZ, 20 μM) (B) Phasor signatures of both reporters in isolation (Ca^2+^ reporter 1 green cursor) (Ca^2+^ reporter 2 yellow cursor) in the presence of Fz or CTZ (20 μM). (C) Phasor signatures of the mixture of the cells expressing each probe in (B) in the presence of Fz and CTZ (20 μM). (D) Images of the sensor-expressing HEK (smaller) and HeLa (larger) cells from (A), either spread out or in contact. Cells were treated with Fz and CTZ (20 μM). Pseudo color represents the intensity and phase (in radians). The phasor plot displays the locations of individual cells based on their phase and modulation characteristics. Scale bars, 25 μm. A total of 20 frames were collected per slice, and the phasor locations were computed. Data are representative of *n* = 3 replicate studies.

We hypothesized that bioluminescent phasor analysis could distinguish unique sensors in a single image. This capability could enable studies of calcium dynamics across multiple cell types simultaneously, providing new insights into multicellular interactions. Sensors could also be more reliably used to report on dynamic calcium flux in different subcellular compartments. Simultaneous readouts are extremely challenging with conventional technologies, as the often subtle and rapid changes in spectral outputs cannot be easily discerned. To test bioluminescent phasor in this context, we first examined the phasor outputs of three distinct calcium reporters, NL(Ca^2+^), CaFluxVTN, and GeNL(Ca^2+^)_520 (calcium reporters 1–3, respectively, Figure 6A). NL(Ca^2+^) comprises a Renilla luciferase (RLuc) donor and Venus fluorescent protein acceptor flanking a well-established calmodulin-M13 domain.^43^ GeNL(Ca^2+^)_520 is constructed similarly, except with NanoLuc as the donor.^19^ In both cases, Ca^2+^ binding results in a conformational shift, resulting in enhanced BRET (Venus emission). HEK293 and HeLa cells were engineered to express the three different reporters, and phasor positions were determined (Figure S6F). Subsequent experiments were conducted with NL(Ca^2+^) and CaFluxVTN given their more resolvable phasor positions.

When imaged separately, the two Ca^2+^ biosensors exhibited distinct and reproducible phasor signatures (Figure 6B). Notably, the phasor position also varied depending on the cell type, reflecting differences in cell-specific calcium signaling (Figure S7). When the CaFluxVTN- and NL(Ca^2+^)-expressing cells were mixed together, both phasor signatures remained clearly resolvable, demonstrating that the platform is suitable for reliable multiplexed biosensing for a single sample (Figure 6C). Interestingly, an increased BRET signal was observed for CaFluxVTN-expressing cells in the mixed population, likely reflecting altered signaling activity upon co-culture.^44^^,^^45^^,^^46^ This result also motivated further investigation using single-cell analyses. Mixed populations of reporter 1 (HEK cells expressing NL(Ca^2+^)) and reporter 2 (HeLa cells expressing CaFluxVTN) were imaged under conditions where cells were either dispersed or in contact (Figure 6D). Phase color maps revealed shifts corresponding to cell proximity, with green indicating higher BRET efficiency when cells were in close contact, and blue indicating lower BRET efficiency when dispersed. These phase shifts enabled a clear differentiation between reporter populations within a single imaging frame. The phase characteristics, in this example, can be used to distinguish individual cells based on their spatial organization and interaction state. Such spectral differences are difficult to assign to distinct reporters using conventional emission assays (Figure S7A) or spectral filtering, given the overlapping spectra. Altered sensor activity was corroborated when CaFluxVTN-expressing HeLa cells were incubated with different numbers of unlabeled HEK cells (Figures S7B and S7C). An increase in BRET efficiency in this case—measured as the acceptor (533 nm) to donor (460 nm) emission ratio—was observed with higher proportions of unlabeled cells and with the increasing confluency (Figure S7D).

## Discussion

In this work, we expanded the scope of bioluminescent phasor technology. This platform offers a streamlined approach for distinguishing overlapping spectra by transforming the emission data at each pixel into spectral phasors. The technique is notably different from other methods for resolving multicolored reporters, including spectral unmixing.^47^^,^^48^^,^^49^ No prior knowledge of mixture composition is required, and real-time multiplexing can be performed without the need for complicated filter switching. The bioluminescent phasor approach is, thus, well suited for applications with low-level light emitters, where imaging speed is paramount.

In this work, we introduced an expanded panel of phasor signatures to enable multiplexing of additional bioluminescent probes. We evaluated the LumiFluors and variants of Antares. All characterized probes could be readily resolved from each other and their Nano-lantern counterparts. Notably, many orange-to-red emitters were readily multiplexed using bioluminescent phasor imaging. These reporters typically have a poor donor-acceptor overlap, and thus emit few photons that can be detected with standard wavelength filtering.^26^^,^^27^ Despite the suboptimal energy transfer profiles, the reporters are distinguishable through phasor analysis, eliminating the need for time- and resource-intensive efforts to enhance their light outputs. Even exceptionally small changes (<10 nm) in emission spectra were sufficient for multiplexed imaging. The reporters cataloged add to the growing list of bioluminescent probes that can be used for multicomponent readouts.

We further applied bioluminescent phasor technology to imaging tissues from mouse models. Bioluminescence is uniquely suited for sensitive imaging applications in organs and whole organisms, where supplying exogenous light sources is difficult and often hindered by autofluorescence. We demonstrated that single cells expressing reporters can be analyzed at varying depths, with reporter detection achievable in excised tumors *ex vivo* and also *in vivo*, using two independent mouse models.

In another key advance, we applied bioluminescent phasor analysis to examine established sensors. Readouts from both protease- and metabolite-responsive reporters could be successfully tracked in cultured cells and within tissue matrices. Multiple calcium sensors with overlapping emission spectra could also be distinguished within cell mixtures, setting the stage for more refined studies of cell signaling. Even subtle changes in emission profiles could be detected in populations of metabolite-responsive cells, and signals could be acquired serially at multiple depths—critical for longitudinal studies where light emission decays over time. It should be noted, though, that the bioluminescent reporters must be spatially separated to be reliably resolved based on phase only.

Collectively, these results establish that bioluminescent phasor imaging can distinguish multiple biosensor populations in real time, even when their emission spectra are highly similar. This multiplexing capability is especially valuable for studying complex interactions such as tumor-immune cell models, where distinct signaling pathways must be monitored simultaneously within a shared environment. Future work will continue to broaden the scope of bioluminescent phasor technology. More sensitive cameras will enable imaging of dimmer bioluminescent probes and substrates. Alternative imaging setups, such as upright microscopes, may be more suitable for *in vivo* imaging. Imaging in these complex environments also requires further considerations. For example, wavelengths <650 nm are absorbed by heme and other biomolecules.^50^ Improved probes and detection platforms will expand the types of dynamic cellular behaviors that can be visualized, providing new insights into physiology and disease.

### Limitations of the study

Bioluminescent phasor has some limitations for direct application in thick tissues and whole animals. Densely labeled samples can complicate multiplexed imaging, and reliable discrimination based solely on phase requires spatial separation of the bioluminescent sources. Sensitive imaging *in vivo* also requires bright, red-emitting probes. Most luciferase-luciferin combinations emit sub-optimally in the tissue-penetrant region—highlighted by the void in the bottom right quadrant of the phasor plot.

Scattering and out-of-focus light also become more challenging in heterogeneous environments. Additionally, vertical microscopes are more difficult to use for capturing signals from mouse xenografts. Future studies with thick samples would benefit from an upright microscope, as this configuration provides better access to the sample surface with the objective lens, enabling deeper imaging into the tissue while minimizing light scattering. In addition, since maximizing photon detection is always critical in bioluminescence imaging, this study employed a TIRF-equipped system with a high numerical aperture (NA, 1.49) to enhance photon collection under low-light conditions. This setup differs from standard widefield systems, and future work will assess reporter performance across alternative imaging platforms.

Another limitation with bioluminescent phasor is the need for two reference cameras, adding cost and complexity. The 2-way splitter on each camera reduces photon capture by 25%. While this allows for simultaneous sin/cos data acquisition, it prevents real-time phasor transforms, requiring post-processing and increasing the analysis time and computational load. Moreover, the current design does not support other modalities, such as fluorescence, for easy referencing, which could be useful in some contexts for improved signal interpretation. Additionally, the focus and field of view (FoV) cannot be adjusted in real time, limiting flexibility during dynamic imaging experiments. These limitations can be addressed with more advanced bioluminescent phasor hardware and software, but they restrict the current system’s responsiveness and versatility in some experimental conditions.

## Resource availability

### Lead contact

Requests for further information and resources should be directed to and will be fulfilled by the lead contact, Jennifer A. Prescher (jpresche@uci.edu).

### Materials availability

This study did not develop new materials or reagents.

### Data and code availability

• Data generated for this study are either reported in the paper or available from the lead contact upon request.
• Code used to replicate the analyses reported in this study is available at Github: https://github.com/LorenzoScipioni/Phasor---Bioluminescence (https://doi.org/10.5281/zenodo.18405269).
• Any additional information required to re-analyze the data reported in this study is available from the lead contact upon request.

## Acknowledgments

This research was supported by an Allen Distinguished Investigator Award, a 10.13039/100017023Paul G. Allen Frontiers Group advised grant of the 10.13039/100000952Paul G. Allen Family Foundation (to J.A.P. and M.A.D.), the 10.13039/100014989Chan Zuckerberg Initiative (to J.A.P. and M.A.D.), the US National Institutes of Health (grant no. R01 GM107630 to J.A.P. and grant no. R01 DE030123 to A.L.A.), the UNC University Cancer Research Fund (to A.L.A.), a Beall Innovation Award (to J.A.P.), and startup funding support from the 10.13039/100009164Moffitt Cancer Center (to A.L.A.). C.K.B. was supported by the UCI Physical Sciences Machine Learning NEXUS program. We thank members of the Laboratory of Fluorescence Dynamics (LFD, UCI) for discussion. Some experiments were performed at the Laser Spectroscopy Laboratories (LSL) at UC Irvine.

## Author contributions

L.P.H., C.K.B., L.S., G.T., M.A.D., and J.A.P. conceived the project idea; L.P.H., C.K.B., L.S., G.T., Z.R.T., M.A.D., and J.A.P. developed the methodology; L.P.H., C.K.B., L.S., G.T., Z.R.T., C.G., M.A.D., and J.A.P. designed experiments; L.P.H., C.K.B., L.S., G.T., Z.R.T., K.P.-S., and B. L. performed experiments; L.P.H., C.K.B., L.S., G.T., Z.R.T., and C.G. conducted imaging analyses; L.P.H., C.K.B., L.S., and J.A.P. wrote the initial manuscript draft. All authors participated in manuscript reviewing and editing.

## Declaration of interests

The authors declare no competing interests.

## STAR★Methods

### Key resources table

**Table:** 

REAGENT or RESOURCE	SOURCE	IDENTIFIER
**Bacterial and virus strains**		

*Escherichia coli*: One Shot Top 10; Chemically Competent Cells	Invitrogen	Lot # 2412803; Ref #C404010

**Biological samples**		

Mouse-derived xenografts	University of North Carolina at Chapel Hill	–

**Chemicals, peptides, and recombinant proteins**		

ReNL	Addgene	89536
Ca2+ carbonate dihydrate	Fisher Scientific	CAS Number: 10035-04-8
EGTA	Sigma Aldrich	CAS Number: 67-42-5
STS	VWR	CAS Number: 62996-74-1
Furimazine	Promega	Catalog number: N1110
Puromycin	–	–
Coelenterazine	GoldBio	Catalog Number: CZ2.5 CAS Number: 55779-48-1
Catalase from bovine liver	Sigma Aldrich	CAS No. 9001-05-2

**Experimental models: Cell lines**		

Human: HEK293 (kidney)	ATCC	CRL-1573
Human: HeLa (uterus/cervix)	ATCC	CCL-2 ™
Human: MDA-MB231 (Breast)	ATCC	HTB-26
Human: UM-HMC-1(Salivary gland)	Laboratory of Jacques E. Nör (University of Michigan, Ann Arbor, MI)	RRID: CVCL_Y473

**Experimental models: Organisms/strains**		

Mouse: (Crl:NU(NCr)-*Foxn1*^*nu*^	Charles River	Strain code: 490
Mouse: (Nu/Nu)	Animal Studies Core at the University of North Carolina at Chapel Hill	RRID: IMSR_JAX:002019

**Oligonucleotides**		

Primers for pLenti cloning (Materials and Methods Section)	–	–

**Recombinant DNA**		

NanoLuc	Yao et al.^33^	Addgene: 183045

CeNL	Yao et al.^33^	Addgene: 208839
GeNL	Yao et al.^33^	Addgene: 208841
YeNL	Yao et al.^33^	Addgene: 208849
OeNL	Yao et al.^33^	Addgene: 208850
CeNLuc	Musicant et al.^24^	Addgene: 208838
GpNLuc	Schaub et al.^25^	Addgene: 208840
OgNLuc	Schaub et al.^25^	Addgene: 208851
dKenLuc	Labra et al.^26^	Addgene: 208856
KaNLuc	Labra et al.^26^	Addgene: 208855
LumiScarlet	Yeh et al.^14^	Addgene: 208854
C9-BRET	Den Hamer et al.^40^	Addgene: 98289
CafluxVTN	Yang et al.^41^	Addgene: 83926
Nano-Lantern(Ca2+)	Saito et al.^43^	Addgene: 51982
GeNL-Ca520	Suzuki et al.^19^	Addgene: 85204
ReNL	Suzuki et al.^19^	Addgene: 89536

**Software and algorithms**		

ImageJ	–	https://imagej.nih.gov/ij/
MATLAB algorithm	–	https://github.com/LorenzoScipioni/Phasor---Bioluminescence
IVIS software: Living Image (version 4.5.4)	PerkinElmer	–
Google Colab algorithm	–	–
GraphPad/Prism 10	GraphPad Software	https://www.graphpad.com/
Adobe Illustrator 2025	–	–

### Experimental model and study participant details

#### Mammalian cell culture

HeLa (ATCC), HEK293 (ATCC), MDA-MB231 (ATCC), and Lenti X-293T (Takara no. 632180) cells were cultured in complete medium consisting of DMEM (Corning) containing 4.5 g/L glucose and 2 mM L-glutamine, supplemented with 10% (v/v) fetal bovine serum (FBS, Life Technologies), penicillin (100 U/mL), and streptomycin (100 μg/mL, Gibco). Cells were incubated at 37°C in a 5% CO_2_ humidified chamber and serially passaged using trypsin (0.25% in HBSS, Gibco). Cells were counted using an automated cell counter (Countess II, Invitrogen). All cell lines used in this study, including HeLa, HEK293, MDA-MB231, and Lenti X-293T, are derived from female human tissues, and their sex was verified based on the origin of the cells.

#### *In vivo* cell transplants

##### Cohort #1

All animal experiments were conducted in 6–8-week-old male and female athymic nude mice (Nu/Nu) obtained from the Animal Studies Core at the University of North Carolina at Chapel Hill. Mice were group housed under the Division of Comparative Medicine (Chapel Hill, NC, USA), in compliance with institutional and national guidelines for the care and use of laboratory animals. Specifically, 6–8-week-old littermates of the same sex were group housed and randomly assigned to experimental groups for subcutaneous tumor cell transplantation.

UM-HMC-1 cells stably expressing one of three reporters (CeNLuc, YeNL, or LumiScarlet) were grown under 0.5 μg/mL puromycin selection until they reached 80–90% confluence and then dissociated using TrypLE Express (Thermo Fisher Scientific, cat# 12604039). For each tumor, 9 × 10^5^ cells were pelleted at 600 ×*g* for 5 min, kept on ice, and resuspended in ice-cold Hank’s Balanced Salt Solution (HBSS; Gibco, cat# 14175-095). Five unique xenografts were prepared: (1) CeNLuc only, (2) YeNL only, (3) LumiScarlet only, (4) an equal-proportion mixture (1:1:1), and (5) a mixed-proportion cocktail consisting of 10% CeNLuc, 80% YeNL, and 10% LumiScarlet. Each suspension was thoroughly mixed 1:1 with ice-cold Cultrex Basement Membrane Extract (R&D Systems, cat# 343200501P), and 100 μL of this final preparation was injected subcutaneously into each of the two dorsal lateral hind flank regions. Individual mice received duplicate injections of a single xenograft type, generating ten total tumors across the five xenograft groups.

Tumor growth was monitored by measuring length and width with a caliper, and tumors were resected once they reached approximately 250 mm^3^. Tumor volume was determined by applying a modified ellipsoidal formula: tumor volume = 1/2(length × widthˆ2).

##### Cohort #2

Female athymic nude mice (Crl:NU(NCr)-Foxn1nu) were obtained from Charles River. All animal procedures were approved by the Institutional Animal Care and Use Committee (IACUC) of the University of California, Irvine, in compliance with the National Institutes of Health guidelines, under protocol number AUP-21-137. All mice used were female; no sex preference was applied, and selection was based on animal availability within the colony. Mice were maintained on a 12 h light/dark cycle at 25°C with humidity maintained between 30% and 70%, and all procedures were performed during the light portion of the cycle. Mice were anesthetized using isoflurane (1–2%) and received subcutaneous ventral injections of MDA-MB231 cells (ATCC, 2 × 10^6^) expressing enhanced Nano-Lanterns (or LumiScarlet) suspended in 200 μL Matrigel (Corning 354234). After one week, mice received an intraperitoneal injection of Fz (100 μM, 100 μL total volume, 1:20 dilution in PBS) and were subsequently imaged.

### Method details

#### General cloning methods

Polymerase chain reaction (PCR) methods were used to amplify and isolate all genetic elements. FLAG tagged versions of OeNL and LumiScarlet were cloned as previously described.^33^ FLAG tagged versions of Nluc, CeNL, GeNL, YeNL, CeNLuc, OgNLuc, GpNLuc, dKeNLuc, and KaNLuc were cloned into the pLenti backbone as follows:

The reporters were amplified and a 5′ *Xba*I site and N-term FLAG tag, and a 3′ *Bam*HI site added using the following primers:

Nluc forward primer:

5ʹ-ctcctcTCTAGAATGGACTACAAGGACGACGATGACAAGGTCTTCACACTCGAAGA.

TTTCGTTGGG-3ʹ.

CeNL, YeNL, CeNLuc, OgNLuc, and GpNLuc forward primer:

5ʹ-ctcctcTCTAGAATGGACTACAAGGACGACGATGACAAGGTGAGCAAGGGCGAGG.

AG-3ʹ.

GeNL forward primer:

5ʹ-ctcctcTCTAGAATGGACTACAAGGACGACGATGACAAGGTGTCCAAGGGCGAAG.

AGGACAA-3ʹ

dKeNLuc forward primer:

5ʹ-ctcctcTCTAGAATGGACTACAAGGACGACGATGACAAGGTGAGTGTGATCGCTAA.

ACAAATGACCTAC-3ʹ.

KaNLuc forward primer:

5ʹ-ctcctcTCTAGAATGGACTACAAGGACGACGATGACAAGAGCGAGCTGATTAAGGA.

GAACATGCA-3ʹ.

Universal reverse primer:

5′-ctcctcGGATCCTTACGCCAGAATGCGTTCGCAC-3′

All PCR reactions (unless otherwise stated) were performed in a BioRad C3000 Thermocycler using the following conditions: 1) 95°C for 3 min, 2) 95°C for 30 s, 3) T_m_ of primers for 30 s, 4) 72°C for 3 min, repeat steps 2–4 twenty to thirty-four times, then 72°C for 5–10 min, and hold at 12°C until retrieval from the thermocycler.

The pLenti vector (Addgene no. 73582) was linearized via digestion with restriction enzymes *Xba*I and *Bam*HI (NEB #R0145 and R0136, respectively). The resulting linearized vector was dephosphorylated using recombinant Shrimp Alkaline Phosphatase (NEB, cat #M0371). The digested/dephosphorylated plasmid was column purified (Macherey-Nagel PCR clean up kit, cat. #: 740609). The digested vector backbone and insert (1:6 ratio) were ligated overnight using T4 DNA ligase (NEB, cat#; M202) and transformed into chemically competent *E. coli* Stable3 cells (NEB, cat#; C304).

CafluxVTN (Venus-TnC-NanoLuc) and caspase-9 BRET sensor (mNeonGreen-LEHD-NanoLuc) were amplified using primers. Amplified genes were ligated into destination vectors via Gibson assembly.^51^

CafluxVTN forward primer:

5′ atccacgctgttttgacctccatagaagacaccgactctagaatggtgagcaagggcgag 3′

CafluxVTN reverse primer:

5′ ggtcgaccactggtcgacgcgttaactagtccggatcccgttacgccagaatgcgttcgc 3′

Caspase-9 BRET forward primer:

5′ atccacgctgttttgacctccatagaagacaccgactctagaatggtctccaagggggaa 3′

Caspase-9 BRET reverse primer:

5′ ggtcgaccactggtcgacgcgttaactagtccggatcccgtcaagccagaatcctttcgc 3′

PCR reactions were performed in a BioRad C3000 thermocycler using the following conditions: 1) 95°C for 3 min, 2) 95°C for 30 s, 3) −1.2°C per cycle starting at 72°C for 30 s, 4) 72°C for 30 s, repeat steps 2–4 ten times, 5) 95°C for 3 min, 6) 95°C for 30 s, 7) 60°C for 30 s, 8) 72°C for 2 min repeat steps 6–8 twenty times, then 72°C for 5 min, and hold at 12°C until retrieval from the thermocycler. The pLenti vector (Addgene no. 73582) was linearized via digestion with restriction enzymes *Xba*I and *SpeI* (NEB #R0145 and R3133, respectively). The digested vector backbone and insert (1:5 ratio) were ligated using Gibson assembly. Gibson assembly conditions were: 50°C for 60 min and held at 12°C until retrieval from the thermocycler. Plasmids were transformed into TOP10 *E. coli* cells using the heat shock method. After incubation at 37°C for 18–24 h, colonies were picked and expanded overnight in 5 mL LB broth supplemented with ampicillin (100 μg/mL). DNA was extracted from colonies using a Zymo Research Plasmid Mini-prep Kit (D4211). DNA was subjected to restriction enzyme digestion to confirm gene insertion. Positive hits were confirmed with Sanger sequencing.

Plasmids encoding teLuc (Addgene plasmid no. 100026) and Antares2 (Addgene plasmid no. 100027) were gifts from H.-w. Ai. The plasmid encoding Antares (Addgene plasmid no. 74279) was a gift from M. Lin. The plasmid encoding CalfluxVTN was purchased from Addgene (plasmid no. 83926). The plasmid encoding caspase-9 BRET Sensor was purchased from Addgene (plasmid no. 98289). Each plasmid was transformed into TOP10 competent *E. coli* cells. The transformants were plated on agar plates containing carbenicillin (50 μg/mL). Colonies containing the genes of interest were expanded overnight in 5 mL LB broth containing ampicillin (50 μg/mL). Plasmid DNA was extracted using a Zymo Research Plasmid Mini-prep Kit and concentrations were measured using a Nanodrop 2000c Spectrophotometer (Thermo Scientific).

#### Plasmids

**Table:** 

reporter	Features	Plasmid Vector	Promoter	Expression in	Addgene#
NanoLuc	FLAG - Nluc - SV40 - PuroR - WPRE - AmpR	pLenti 3^rd^ gen	CMV	Mammalian	183045
CeNL	FLAG - mTurquoise2 - Nluc - SV40 - PuroR - WPRE - AmpR	pLenti 3^rd^ gen	CMV	Mammalian	208839
GeNL	FLAG - mNeonGreen - Nluc - SV40 - PuroR - WPRE - AmpR	pLenti 3^rd^ gen	CMV	Mammalian	208841
YeNL	FLAG - Venus - Nluc - SV40 - PuroR - WPRE - AmpR	pLenti 3^rd^ gen	CMV	Mammalian	208849
OeNL	FLAG - mKusabiraOrange - Nluc - SV40 - PuroR - WPRE - AmpR	pLenti 3^rd^ gen	CMV	Mammalian	208850
CeNLuc	FLAG - mCerulean3 - Nluc - SV40 - PuroR - WPRE - AmpR	pLenti 3^rd^ gen	CMV	Mammalian	208838
GpNLuc	FLAG - eGFP - Nluc - SV40 - PuroR - WPRE - AmpR	pLenti 3^rd^ gen	CMV	Mammalian	208840
OgNLuc	FLAG - LSSmOrange - Nluc - SV40 - PuroR - WPRE - AmpR	pLenti 3^rd^ gen	CMV	Mammalian	208851
dKenLuc	FLAG - dKeima - Nluc - SV40 - PuroR - WPRE - AmpR	pLenti 3^rd^ gen	CMV	Mammalian	208856
KaNLuc	FLag - LSSmKate2 - Nluc - SV40 - PuroR - WPRE - AmpR	pLenti 3^rd^ gen	CMV	Mammalian	208855
LumiScarlet	FLAG - mScarlet - Nluc - SV40 - PuroR - WPRE - AmpR	pLenti 3^rd^ gen	CMV	Mammalian	208854
C9-BRET	mNeonGreen-LEHD-Nluc-- SV40 - PuroR - WPRE - AmpR	pLenti 3^rd^ gen	CMV	Mammalian	98289 (pcDNA 3.1 (+))
CafluxVTN	Venus-TnC-Nluc - SV40 - PuroR - WPRE - AmpR	pLenti 3^rd^ gen	CMV	Mammalian	83926 (pRSETb)
Nano-Lantern(Ca2+)	VenusΔC10-NRluc8ΔN3-CAM-M13-104-2G-CRluc8-SV40-AmpR	pCDNA3	CMV	Mammalian	51982
GeNL-Ca520	mNeonGreenΔC10-NlucΔ5-CAM-M13-Nluc-SV40-AmpR	pCDNA3	CMV	Mammalian	85204
ReNL	6xHIS - ReNL -- AmpR	pRSETb	T7	Bacterial	89536

#### Synthetic reagents

All reagents purchased from commercial suppliers were of analytical grade and used without further purification.

#### Transient transfections

Cells (2.5 x 10^5^) were seeded in 12-well sterile dishes (Corning). Transfections were performed the following day when cells were 75–80% confluent, using luciferase constructs and Lipofectamine 3000 (Thermo Fisher) according to the manufacturer’s instructions. Approximately 16 h post-transfection, cells were lifted with trypsin and plated (5 x 10^5^ cells) on tissue-culture treated 8-well chambered coverslips (μ-Slide 8 Well ibiTreat, Ibidi). In some cases, the coverslips were coated with 5 mg/cm^2^ fibronectin human plasma (Millipore Sigma) according to the manufacturer’s instructions.

#### Viral transductions

Viruses were packaged as previously described.^24^ Briefly, FLAG-tagged reporter plasmids (in pLenti backbones, described above) were co-transfected with the VSV-G envelope plasmid and d8.2 gag/pol helper plasmids into Lenti X-293T cells (seeded in 10 cm dishes). Transfections were performed using 1 mg/mL polyethyleneimine (PEI, VWR #BT129700). A total of 1.5 μg VSV-G, 5 μg d8.2, and 6 μg FLAG-pLenti plasmids were mixed in 500 μL OptiMEM (LifeTech #1158021) and vortexed. In a separate tube, 25 μL PEI (2 μL PEI/μg DNA) was added to 475 μL OptiMEM and vortexed. Both solutions were incubated at room temperature for 5 min, then combined and incubated for an additional 20 min. The resulting mixture was added dropwise to the seeded Lenti X-293T cells.

The following day, the cell culture medium was replaced with DMEM supplemented with 1x NaPyr, 10 mM HEPES, 1X GlutaMAX, and 1X PSG (no FBS). Two days later, the media was collected, filtered through a 0.45 μm PVDF membrane, and the viral particles were concentrated by ultracentrifugation (100,000 ×*g* for 2 h at 4°C using an SW28 rotor on a Beckman L8-80M centrifuge) into a sucrose cushion. The concentrated virus was resuspended in cold PBS and either stored at −80°C or used immediately.

#### General bioluminescence and fluorescence imaging

Luminometer readings were performed in black, flat-bottom 96-well plates (Greiner Bio-One). Samples were plated and measured in triplicate unless otherwise stated. Bioluminescence measurements were acquired with a Tecan Spark M10 multimode microplate reader. Data were collected using an integration time of 1000 ms unless otherwise stated. Light emission was measured in counts/s. Fluorescence measurements were acquired on a Tecan Spark M10 multimode microplate reader for CeNLuc (excitation 420 nm, emission 490 nm, 20 nm bandwidth), YeNL (excitation 485 nm, emission 535 nm, 20 nm bandwidth), and LumiScarlet (excitation 570 nm, emission 615 nm, 20 nm bandwidth). Mice were imaged in a light-proof chamber using an IVIS Lumina (Xenogen) system with a supercooled CCD camera chilled to −85°C. The stage was kept at 37°C during imaging. Living Image software was used to control the instrument and measure photon flux values from defined regions of interest. Exposure times were set to 1 s in all experiments. All data were exported to Microsoft Excel or GraphPad Prism (version 7.0c for Macintosh, GraphPad Software, La Jolla, CA, USA; www.graphpad.com) for further analyses.

#### Protease biosensor experiments

HeLa cells stably expressing the caspase 9 BRET sensor (C9-BRET) (2 x 10^5^) were plated in triplicate into black 96-well plates (Greiner Bio-One). After 18 h, cell media was exchanged for phenol red-free DMEM (Gibco FluoroBrite DMEM, A1896701) containing Fz (50 μM) and 5 μM STS (CAS Number: 62996-74-1, VWR). Luminescence spectra were analyzed over time using a luminometer (Tecan Spark M10 multimode microplate reader).

#### Calcium biosensor experiments

HEK293 cells were transiently transfected with CafluxVTN BRET sensor (pLenti). After 18 h. the cells were lifted, spun down (0.5 g-force), 5 min) x 2, and the media was replaced with a buffer containing 1 = 10 mM EGTA, 100 mM KCl, 30 mM MOPS, pH 7.2. The cells were lysed using a 30 gauge syringe and then supplemented with increasing concentrations of Ca^2+^. Luminescence spectra were measured on a luminometer (Tecan Spark M10 multimode microplate reader).

HeLa cells stably expressing CafluxVTN BRET sensor (pLenti) (2 x 10^5^) were plated in triplicate into black 96-well plates (Greiner Bio-One). After 18 h, cell media was exchanged for Ca^2+^-free DMEM (Gibco high glucose, no glutamine, no calcium DMEM, 21068028). Media was then supplemented with 20 μM Fz and 500 μM Ca^2+^ (CAS Number: 10035-04-8) or 10 μM EGTA (CAS Number: 67-42-5, Sigma Aldrich). Luminescence spectra were measured on a luminometer (Tecan Spark M10 multimode microplate reader).

#### Bioluminescent phasor imaging

##### Overview

Bioluminescent phasors were acquired as previously described^33^ on an Olympus IX83 TIRF microscope equipped with two Optosplit II (Cairn) image splitters and used in widefield mode. Briefly, collimated light exiting from the microscope body was split in half by a 50:50 beam splitter and sent to two Optosplit II image splitter devices. In each Optosplit II, the light was further split by a 50:50 beam splitter, half of which passed through a sine or cosine filter (custom made by Auxora) while the other half reached the camera unfiltered. The signal ultimately reached two identical sCMOS cameras (Prime 95b, Photometrics, operated in cooled mode), the acquisition of which was controlled by the μManager software. Unless otherwise stated, all images were recorded with a 10 s integration time and a minimum of 20 frames total were collected per sample. Images were exported and analyzed as described below.

##### Imaging cell samples

HeLa cells were transfected with the appropriate reporter and plated on 8-well Ibidi μ-Slides coated with (10 mg/cm^2^) fibronectin. The next day media was removed and replaced with fresh media (300 μL) containing furimazine (Fz, 3 μL). Five minutes after the media change, the cells were imaged with the TIRF microscope (described above), in widefield mode using a 20x air objective (Olympus UPlanSAPO 20x/0.75) with further 2x magnification.

##### Imaging cells suspended in collagen

HeLa cells expressing YeNL (2 x 10^5^) were suspended in a 2.0 mg/mL collagen Type-1 gel matrix (200 μL, Corning Collagen I, Rat Tail, 100 mg). Following collagen polymerization, media was added (200 μL), and the cultures were incubated at 37°C prior to imaging. The next day, some media was removed (100 μL) and was replaced with media containing Fz (50 μM). Images were acquired with the TIRF microscope as described above using a 10x air objective (Olympus UPlanSApo 10X/0.40).

##### *In vivo* and *ex vivo* imaging

Mice were euthanized and taken to the phasor microscope. Fz (20 μL) was injected directly into the tumor xenografts. Images were acquired with the TIRF microscope as described above using a 10x air objective (Olympus UPlanSApo 10X/0.40). Image analysis was performed as described below on defined tumor region to generate phasor signal.

##### Protease biosensor imaging

HeLa cells stably expressing the caspase 9 BRET sensor (C9-BRET) (2 x 10^5^) were suspended in a 2.0 mg/mL collagen Type-1 gel matrix (as described above) and plated in an 8-well Ibidi μ-Slides coated with (10 mg/cm^2^) fibronectin. The next day, some media was removed (100 μL) and replaced with media containing 50 μM Fz and 50 μM STS (CAS Number: 62996-74-1). Images were acquired with the TIRF microscope as described above using a 20x air objective (Olympus UPlanSAPO 20x/0.75).

##### Calcium biosensor imaging

HEK293 (5 × 10^4^) stably expressing CafluxVTN BRET sensor were plated in 8-well Ibidi μ-Slides coated with (10 mg/cm^2^) fibronectin. After 18 h, cell media was exchanged with Ca^2+^-free DMEM (Gibco, 21068028) and supplemented with 20 μM Fz and either 500 μM Ca^2+^ (CAS Number: 10035-04-8, Fisher Scientific) or 10 μM EGTA (CAS Number: 67-42-5, Sigma Aldrich). Images were acquired with the TIRF microscope as described above using a 20x air objective.

#### Image processing

Images were analyzed as previously reported with some modifications.^33^ In brief, raw data were exported in TIFF format and processed with a custom Python or MATLAB algorithm with the following workflow.

*1. Channel splitting*: The images are split from a single TIFF file in 4 channels corresponding to the sine- and cosine-filtered channels and the appropriate reference channels. The images are cropped in regions of interest (ROIs) of approximately 900 x 385 pixels each and manually registered.
*2. Calibration*: For each day of experiment, a “Dark” image of only the camera noise is acquired as well as a “Bright” image of the transmission lamp (without sine-cosine filters) in order to characterize the camera offset and the effective light splitting in the four channels. The files are cropped to the same ROIs as the experiments and the corrections are applied on a pixel-by-pixel basis.
*3. Processing*: Each intensity image is smoothed by a Gaussian filter with σ = 1 pixel and a variable number of frames (20 for cytoplasmatic reporters) are averaged together. Successively, a global user-defined threshold is set to remove the background contribution and the cells are segmented using the function “bwareafilt” in MATLAB and registered one at the time in all four channels using the function “imregister”.
*4. Phasor transformation and coloring*: The four images are combined to compute the spectral phasor (*G*,*S*) coordinates on a pixel-by-pixel basis. The images are colored according to the bioluminescent reporter.
*5. (Clustering):* For cellular mixtures, clusters in the phasor space were identified using a Gaussian Mixture Model (GMM) clustering in MATLAB using a variable, user-defined number of clusters. The distance between the center of each cluster and the average phasor position of the single reporters was calculated and the cluster was assigned to the closest reporter and color-coded accordingly

#### Phasor transformation

● *Calibration:*
  o *Dark calibration:* Dark calibration is meant to correct the offset and dark noise of the camera. The file needed for the dark calibration is a 100 frames acquisition with the same acquisition parameters as the experiments (10 s integration time) with the camera shutter closed. The dark calibration file is smoothed with a median filter with size 51 x 51 to remove high-frequency noise and an image *I*_*i*,*dark*_ is stored for every channel *i*.
  o *Bright calibration:* The bright calibration is meant to correct the imperfect splitting of light in the four channels, it is needed for a correct phasor position by sine and cosine filters. The file of the bright calibration is a 100 frames acquisition of a homogeneous, bright sample. Here, we use the transmission light from the lamp set at minimum with a 10 ms integration time. The bright calibration file is then smoothed with a median filter with size 51x51 in order to remove high-frequency noise and an image *I*_*i*,*bright*_ is stored for every channel *i*. In order to correct for the uneven intensity splitting in the sine (or cosine) channel and the reference intensity, two parameters are computed:

$$R_{\mathrm{COS}}=\frac{I_{\mathrm{COS},bright-}I_{\mathrm{COS},dark}}{I_{\mathrm{INT}\left(\mathrm{COS}\right),bright-}I_{\mathrm{INT}\left(\mathrm{COS}\right),dark}}{\;R}_{\mathrm{SIN}}=\frac{I_{\mathrm{SIN},bright-}I_{\mathrm{SIN},dark}}{I_{\mathrm{INT}\left(\mathrm{SIN}\right),bright-}I_{\mathrm{INT}\left(\mathrm{SIN}\right),dark}}$$

Where *INT*(*SIN*) and *INT*(*COS*) refer to the reference channel for sine and cosine, respectively.

●*Image correction:* For the experiment files, each channel is corrected as follows

$$I_{\mathrm{INT}\left(\mathrm{SIN}\right)}=I_{\mathrm{INT}\left(\mathrm{SIN}\right)}^{raw}-I_{\mathrm{INT}\left(\mathrm{SIN}\right),dark}$$

$$I_{\mathrm{INT}\left(\mathrm{COS}\right)}=I_{\mathrm{INT}\left(\mathrm{COS}\right)}^{raw}-I_{\mathrm{INT}\left(\mathrm{COS}\right),dark}$$

$$I_{\mathrm{SIN}}=\left(I_{\mathrm{SIN}}^{raw}-I_{\mathrm{SIN},dark}\right)\cdot R_{\mathrm{SIN}}$$

$$I_{\mathrm{COS}}=\left(I_{\mathrm{COS}}^{raw}-I_{\mathrm{COS},dark}\right)\cdot R_{\mathrm{COS}}$$

Where the *raw* denotes the uncorrected images.

● *Phasor transformation:* The (g,s) phasor coordinates are then obtained as follows:

$$g=2\cdot \left(\frac{\frac{I_{\mathrm{COS}}}{I_{\mathrm{INT}\left(\mathrm{COS}\right)}}-F_{\mathrm{COS},\min}}{F_{\mathrm{COS},\max}-F_{\mathrm{COS},\min}}\right)-1$$

$$s=2\cdot \left(\frac{\frac{I_{\mathrm{SIN}}}{I_{\mathrm{INT}\left(\mathrm{SIN}\right)}}-F_{\mathrm{SIN},\min}}{F_{\mathrm{SIN},\max}-F_{\mathrm{SIN},\min}}\right)-1$$

Where *F*_*SIN*,*min*_ and *F*_*SIN*,*max*_ correspond to the minimum and maximum transmission of the sine filter, as obtained from a transmittance measurement performed with a spectrophotometer. Similarly, *F*_*COS*,*min*_ and *F*_*COS*,*max*_ correspond to the minimum and maximum transmission of the cosine filter.

• *Phasor unmixing (2 components):* given the phasor position of the two pure components (*g*_1_,*s*_1_) and (*g*_2_,*s*_2_), the phasor of a combination of the two will lie in position (*g*,*s*) where *g* = *f*_1_*g*_1_+*f*_2_*g*_2_and *s* = *f*_1_*s*_1_+*f*_2_*s*_2_. *f*_1_ and *f*_2_ are the relative contribution of the two pure components to the mixed signal, under the condition *f*_1_+*f*_2_ = 1, that are retrieved by solving the linear system:

$$\left\{ \begin{array}{c} g=f_{1}g_{1}+f_{2}g_{2}\\ s=f_{1}s_{1}+f_{2}s_{2} \end{array}\right.$$

### Quantification and statistical analysis

Quantitative imaging analysis procedures are described in the sections above. All data were exported to Excel and subsequently analyzed and visualized using GraphPad Prism 10 to ensure consistency and accuracy. Data are presented as mean values ±SEM (Standard Error of the Mean) or as otherwise indicated in the figure legends. Boxplots are fully defined in the legends, specifying minima, maxima, center, bounds of the box, whiskers, and percentiles, to allow clear interpretation of the graphical data.

Statistical analyses were performed using Prism 10. Comparisons between two groups were conducted using unpaired, two-tailed t-tests, and multiple group comparisons were performed using Tukey’s multiple comparisons test. Exact *p* values are reported in the figure legends, with statistical significance thresholds defined as follows: (∗) indicates *p* < 0.05, (∗∗) indicates *p* < 0.01, (∗∗∗) indicates *p* < 0.001 and (∗∗∗∗) indicates *p* < 0.0001. The number of independent experimental replicates (n) for each dataset is indicated in the figure legends. No statistical method was used to predetermine sample sizes. Unless otherwise noted, n refers to independent experimental replicates.

## Supplemental information

Document S1. Figures S1–S7 and Tables S1 and S2

Document S2. Article plus supplemental information
