# Supplementary material for: Expanded applications of bioluminescence microscopy with phasor analysis
Source: Cell Rep Methods. 2026 Mar 30;6(4):101344. doi: 10.1016/j.crmeth.2026.101344 (PMC13106971; doi:10.1016/j.crmeth.2026.101344)

Cell Reports Methods, Volume *6*

Supplemental information

Expanded applications of bioluminescence microscopy with phasor analysis

Lila P. Halbers, Caroline K. Brennan, Lorenzo Scipioni, Giulia Tedeschi, Zachary R. Torrey, Kshitij Parag-Sharma, Bryan Labra, Christoph Gohlke, Antonio L. Amelio, Michelle A. Digman, and Jennifer A. Prescher

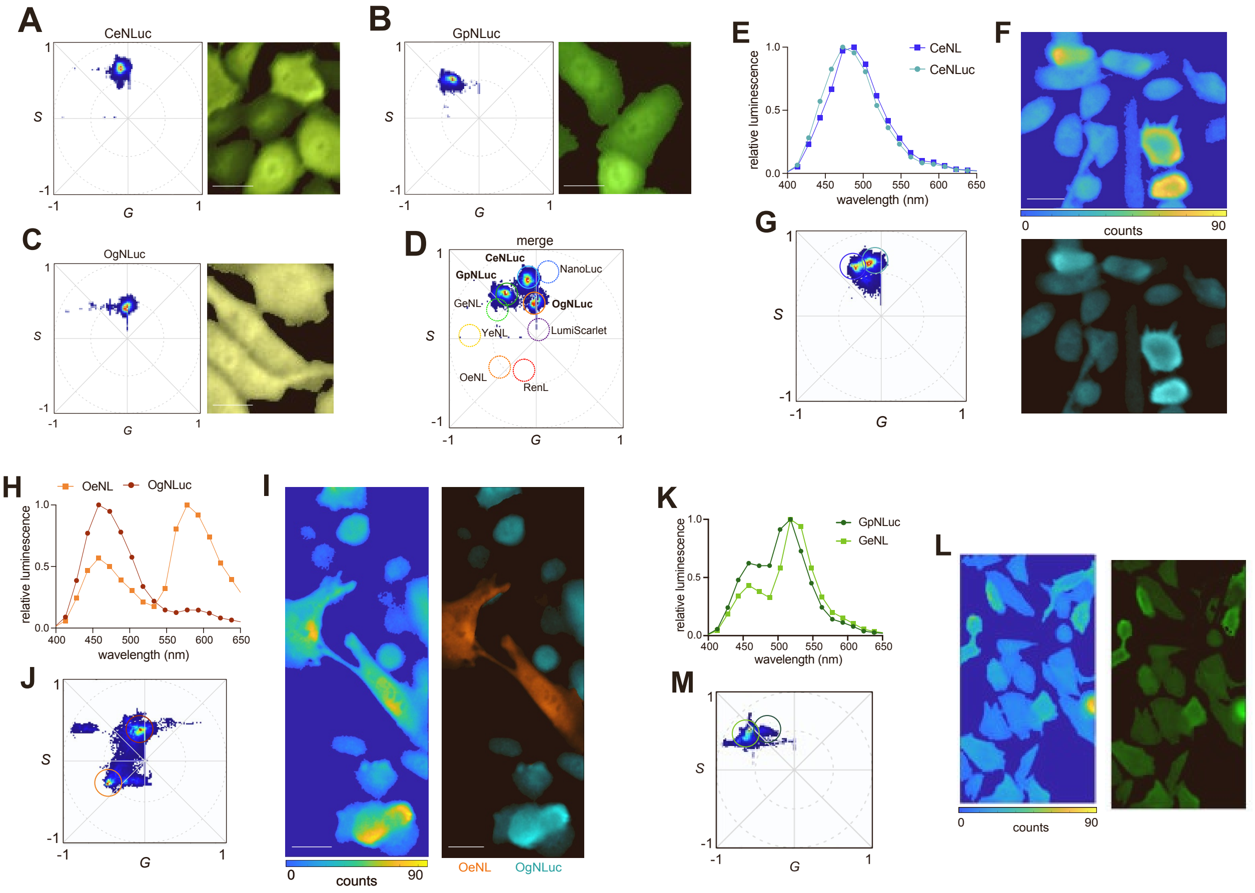

Figure S1: Bioluminescent phasor discrimination of LumiFluor reporters, related to Figure 2. (A–D) Bioluminescent phasor signatures of HeLa cells stably expressing (A) CeNLuc, (B) GpNLuc, or (C) OgNLuc following treatment with furimazine (Fz, 20 μM). (D) Corresponding phasor signatures are distinct from enhanced Nano-lanterns. (E–G) Mixtures of HeLa cells expressing CeNL and CeNLuc. (E) Emission spectra of CeNL and CeNLuc. (F) Mixed populations plated in polymer-coated μ-Slides and imaged following Fz (20 μM) treatment. (G) Pixels were false-colored based on phasor clustering and assigned by reference to single-population phasor signatures. (H–J) Mixtures of HeLa cells expressing OeNL or OgNLuc. (H) Emission spectra of OeNL and OgNLuc. (I) Mixed populations imaged following Fz (20 μM) treatment. (J) Component assignment was performed by comparison to previously reported single populations or those shown in Figure 2E. (K–M) Mixtures of HeLa cells expressing GeNL or GpNLuc. (K) Emission spectra of GeNL and GpNLuc. (L) Mixed populations imaged following Fz (20 μM) treatment. (M) Pixels were assigned to individual reporters based on reference phasor signatures. For all panels, images were acquired using the bioluminescent phasor microscope. A total of 20 frames were collected per sample, and phasor locations were computed from the accumulated data. Each component in the mixture was assigned by referencing to the single populations previously reported.[33] Scale bars = 25 μm.

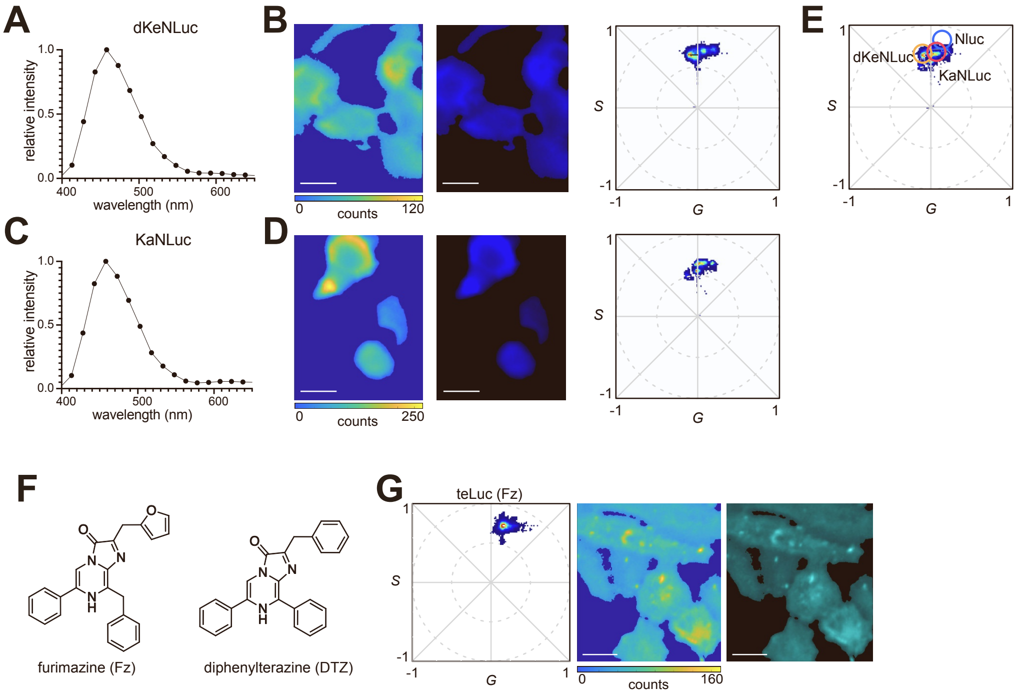

Figure S2: Bioluminescent phasor signatures of red-shifted reporters, related to Figure 2. (A) Bioluminescence emission spectrum of dKeNLuc. (B) HeLa cells stably expressing dKeNLuc following treatment with furimazine (Fz, 20 µM). (C) Bioluminescence emission spectrum of KaNLuc. (D) HeLa cells stably expressing KaNLuc following Fz (20 µM) treatment. (E) Phasor signatures of dKeNLuc and KaNLuc are distinct from NanoLuc (Nluc). (F) Chemical structures of Fz and diphenylterazine (DTZ). (G) HeLa cells transiently expressing teLuc and imaged following Fz (20 µM) treatment. For all panels, images were acquired using the bioluminescent phasor microscope. A total of 20 frames were collected per sample and phasor locations were computed from the accumulated data. Each pixel in the resulting images was color-coded according to the corresponding phasor signature. Scale bars = 25 µm.

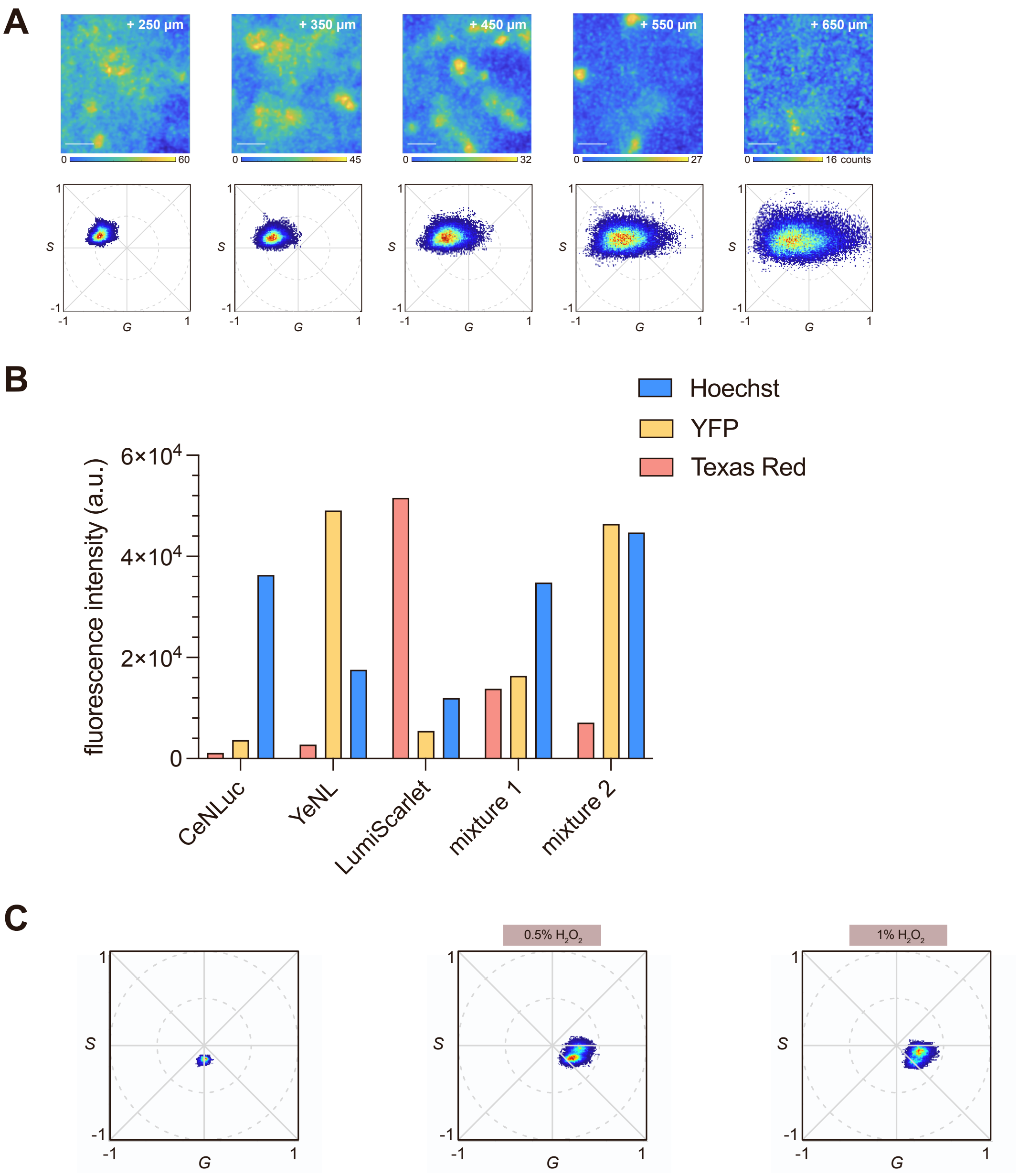

Figure S3: Bioluminescent phasor imaging in tissue environments, related to Figure 3. (A) MDA-MB231 cells expressing YeNL were embedded in a collagen matrix (2.0 mg/mL). Media containing Fz (50 µM) was added to the top of the sample and light emission was recorded with the bioluminescent phasor microscope at various distances from the bottom of the slide using a 10x air objective and 10s/frame integration time. "Counts" represent the intensity above the background. A total of 20 frames were collected per slice and the phasor location was computed. Scale bars = 100 µm. (B) Fluorescence analysis of UM-HMC tumors expressing CeNLuc, YeNL, or LumiScarlet, or mixtures of all three cells (mixtures 1, 2) were resected and analyzed via fluorescence. Each tumor was scanned using pre-set settings (Hoechst: excitation 420 nm, emission 490 nm, 20 nm bandwidth; YFP: excitation 485 nm, emission 535 nm, 20 nm bandwidth; and Texas Red: excitation 570 nm, emission 615 nm, 20 nm bandwidth). (C) Phasor signatures of recombinant ReNL (10 nM) in the presence of 20 µM Fz with the addition 0-1% H_2O_2. Phasors were acquired using the bioluminescent phasor microscope with a 20X air objective and 6 s/frame integration time. A total of 20 frames were collected for each sample and the phasor locations were computed.

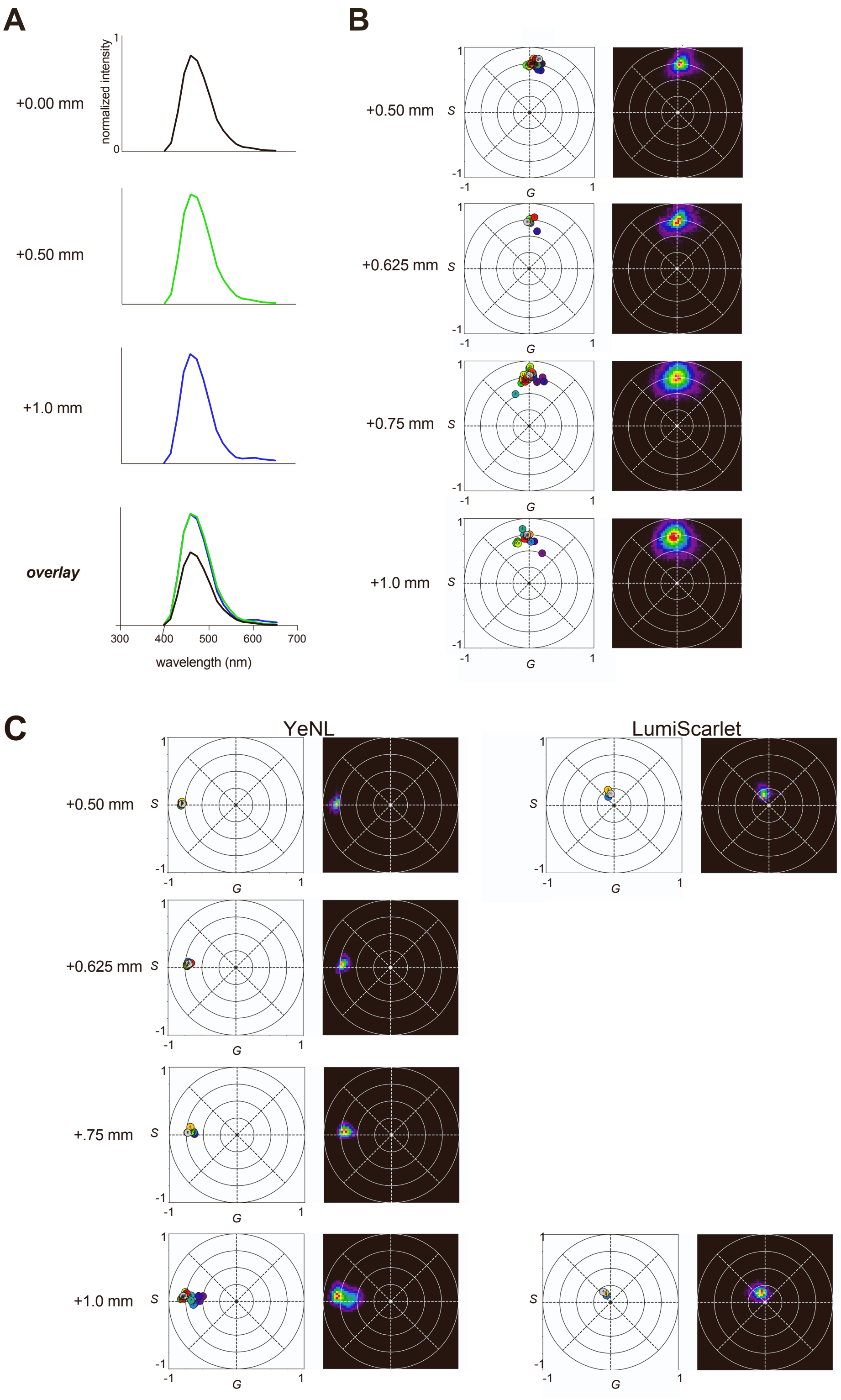

Figure S4: Imaging luciferase reporters through depth, related to Figure 3. (A) HeLa cells expressing NanoLuc were plated on top of varying amounts of collagen (2.0 mg/mL, 0–1 mm thickness). Spectra acquired on a luminometer after Fz (20 μM) addition. (B) Bioluminescent phasor images acquired after adding media containing Fz (50 μM) to the top of each sample. A 20x air objective was used with 10

s/frame integration time. A total of 20 frames were collected per slice and the phasor location was computed. Data are representative of n = 3 replicates. (C) HeLa cells expressing YeNL (left) or LumiScarlet (right) were plated on top of varying amounts of collagen (2.0 mg/mL, 0–1 mm thickness). Bioluminescent phasor images were acquired after adding media containing Fz (50 μM) to the top of each sample. A 20x air objective was used with 10 s/frame integration time. A total of 20 frames were collected per slice and the phasor location was computed. Data are representative of n = 2 replicates.

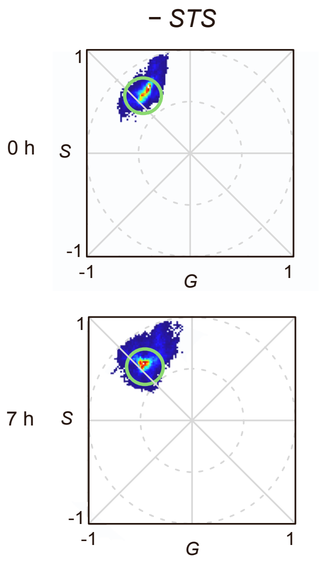

Figure S5: Phasor analysis of caspase activity in the absence of STS, related to Figure 4. HeLa cells expressing C9-BRET were embedded in a collagen matrix (2.0 mg/mL). Media containing Fz (50 μM) without STS was added to the top of the sample, and light emission was recorded on the bioluminescent phasor microscope. A total of 20 frames were collected, and phasor locations were computed.

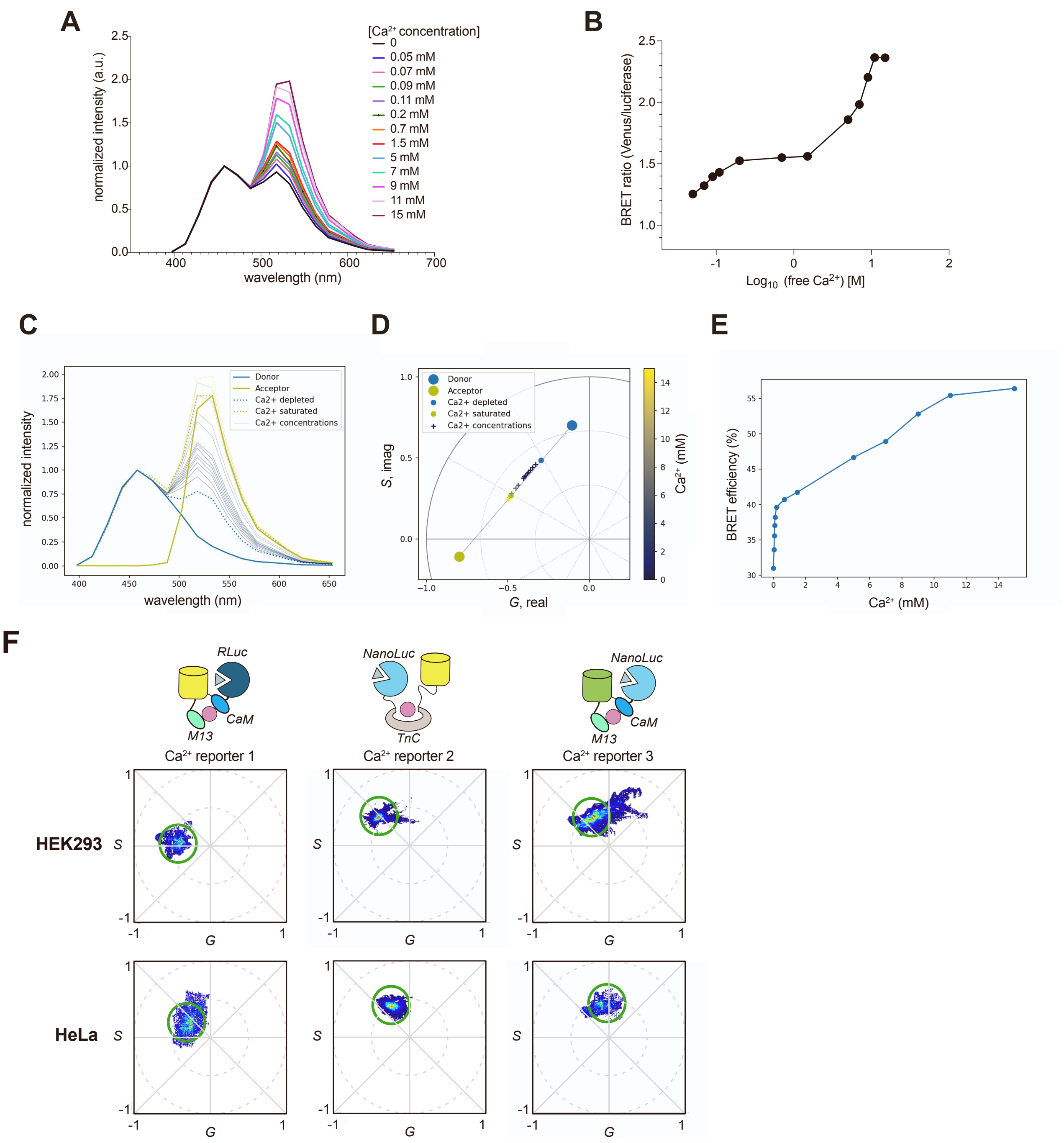

Figure S6: Sensing Ca^{2+} dynamics, related to Figure 5. (A) Bioluminescence emission spectra from lysed CalfluxVTN-expressing cells treated with varying levels of Ca^{2+}. Spectra were recorded on a luminometer. (B) Sigmoidal curve fitting of BRET ratios from samples in (A), using $n = 3$ replicates for exogenously added Ca^{2+} samples, and $n = 10$ replicates for depleted Ca^{2+} sample (C)-(D) Spectra from (A) referenced to emission spectra for NanoLuc and YeNL, showing the full potential dynamic range. The spectra for Ca^{2+} depleted and saturated samples (Figure 5B) were also included. All spectra were then transformed to correlate spectral phasor positions and BRET efficiency measurements with Ca^{2+} concentrations. (F) Phasor signatures of HEK293 and HeLa cells expressing Ca^{2+} reporter 1 (Nano-Lantern(Ca^{2+}) sensor), Ca^{2+} reporter 2 (CafluxVTN), or Ca^{2+} reporter 3 (GeNL(Ca^{2+})_520) in the presence of 20 μM CTZ (reporter 1) or 20 μM Fz (reporters 2 and 3). Phasor outputs were acquired using the bioluminescent phasor microscope with a 20x air objective and 6 s/frame integration time. A total of 20 frames were collected for each sample and the phasor locations were computed. Data are representative of n = 2 replicates.

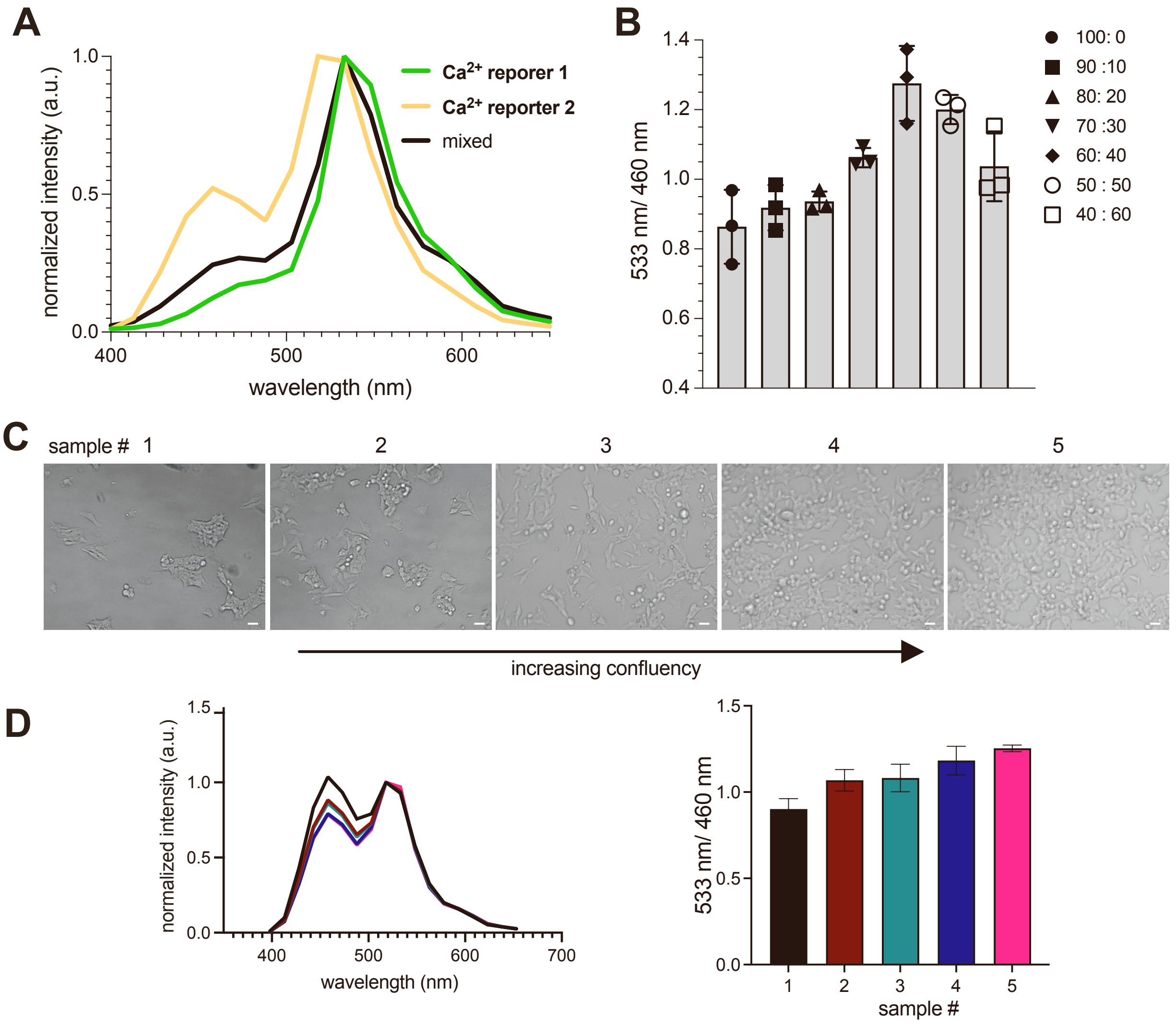

Figure S7: Analysis of calcium reporters using conventional BRET measurements, related to Figure 6. (A) Bioluminescence emission spectra of cultured cell mixtures. HEK293 cells expressing Ca^{2+} reporter 1 (Nano-lantern(Ca^{2+})) were combined with HeLa cells expressing Ca^{2+} reporter 2 (CafluxVTN) (B) HeLa cells stably expressing CafluxVTN were mixed with varying ratios of unlabeled (WT HEK293) cells and analyzed on a luminometer. Light outputs are shown as a ratio of the acceptor emission (Venus, 533 nm) over the donor emission (NanoLuc, 460 nm). Individual (n = 3) replicates are shown, and error bars represent the standard error of the mean. (C) Brightfield images of HeLa cells expressing Ca^{2+} reporter 2 (CafluxVTN) plated 1:1 with unlabeled (WT HEK293). The lowest density sample comprised 5.0×10^3 cells for each cell type, while the highest density sample comprised 5.0×10^4 cells for each cell type. Scale bars = 25 μm. (D) Bioluminescence emission spectra for samples 1-5. Light outputs are shown as a ratio of the acceptor emission (Venus, 533 nm) over the donor emission (NanoLuc, 460 nm). Error bars represent the standard error of the mean for n = 3 replicates.

Table S1: Phasor BRET (%) from sine and cosine filtered imaging, related to Figure 2. Phasor-based BRET efficiencies (%) calculated from sine (S) and cosine (G) filtered data acquired simultaneously across the emission spectrum. Donor-only and acceptor-only phasor coordinates were used to determine the fractional position of the donor-acceptor fusion reporter along the connecting vector, which was then converted to BRET efficiency. Values are listed as “Phasor BRET (%)

Reporter	Acceptor FP	Phasor BRET (%)	Spectral BRET (%)	Difference (phasor − spectral)
CeNL	mTurquoise2ΔC10	99.5	60.31	39.19
GeNL	mNeonGreenΔC10	92.3	95.59	-3.29
YeNL	VenusΔC12	84.9	80.34	4.56
OeNL	mKOκ	65.4	69.59	-4.19
ReNL	tdTomatoΔC9	58.7	65.00	-6.30
CeNLuc	mCerulean3	69.9	54.76	15.14
GpNLuc	eGFP	72.4	61.62	10.78
OgNLuc	LSSmOrange	26.5	12.85	13.65
dKeNLuc	dKeima	4.9	3.65	1.25
KaNLuc	LSS-mKate2	3.3	5.29	-1.99
LumiScarlet (Fz)	mScarlet-I	5.4	38.02	-32.62
Antares	CyOFP1	58.2	55.87	2.33
Antares2 (Fz)	CyOFP1	59.1	56.77	2.33

Table S2: Spectrally derived phasor coordinates (*S*, *G*), related to Figure 2 and STAR Methods. Predicted phasor coordinates (S, G) derived from normalized emission spectra used to calculate "Spectral BRET (%)" in Figure 2A. These values were plotted to generate expected phasor signatures shown below. *Note: S, G coordinates derived from spectra, not from phasor scope measurements.*

**Reporter**	*S*	*G*
NanoLuc	0.73	-0.072
teLuc	0.70	-0.14
CeNL	0.56	-0.35
GeNL	-0.05	-0.75
YeNL	-0.07	-0.53
OeNL	-0.34	-0.065
ReNL	-0.08	-0.012
CeNLuc	0.60	-0.29
GpNLuc	0.40	-0.44
OgNLuc	0.56	-0.13
dKeNLuc	0.70	-0.069
KaNLuc	0.67	-0.064
LumiScarlet	0.22	0.052
Antares	-0.12	0.028
Antares2	-0.13	0.045

*S,G coordinates derived from normalized spectra of reporters

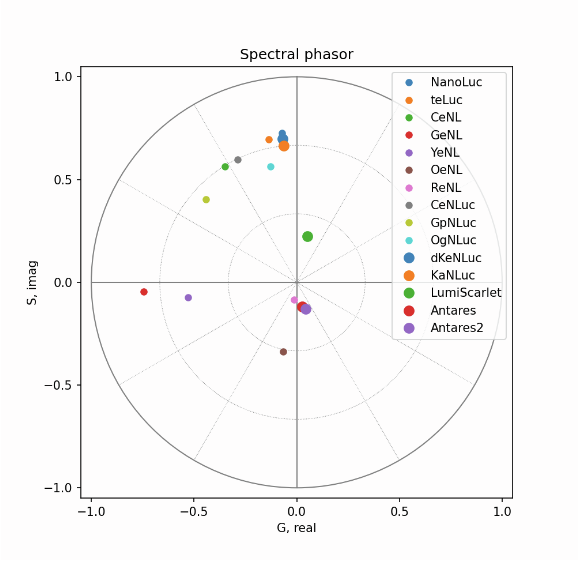

Supplement: Document S1. Figures S1–S7 and Tables S1 and S2 [file mmc1.pdf]
